# Exploring the effect of skim milk on the membrane stability of frozen–thawed Inner Mongolia cashmere goat sperm based on proteomics

**DOI:** 10.3389/fcell.2025.1701830

**Published:** 2026-01-14

**Authors:** Shan-hui Xue, Bing-bing Xu, Wen-ze Li, Jia-xin Zhang, Rui Su

**Affiliations:** 1 College of Animal Science, Inner Mongolia Agricultural University, Hohhot, Inner Mongolia Autonomous Region, China; 2 Sino-Arabian Joint Laboratory of Sheep and Goat Germplasm Innovation, Hohhot, Inner Mongolia Autonomous Region, China; 3 Inner Mongolia Key Laboratory of Sheep and Goat Genetics Breeding and Reproduction, Hohhot, Inner Mongolia Autonomous Region, China

**Keywords:** Inner Mongolia cashmere goat, semen cryopreservation, skim milk, cryodamage, proteomics

## Abstract

**Introduction:**

The cryopreservation of semen from the Inner Mongolia cashmere goat, a valuable dual-purpose breed in China, results in a sharp decline in sperm motility, hindering genetic improvement and germplasm propagation. This study aimed to investigate the protective effects and underlying mechanisms of skim milk as a supplement in a cryopreservation extender.

**Methods:**

Skim milk was added stepwise (2%–3.6%) to an egg yolk–soy lecithin basal extender, with 2.8% identified as the optimal concentration. Tandem mass tag (TMT) quantitative proteomics, coupled with parallel reaction monitoring (PRM) validation, was employed to analyze the proteomic profiles of post-thaw sperm and elucidate homeostatic mechanisms related to sperm membrane stability.

**Results:**

The addition of 2.8% skim milk significantly increased post-thaw sperm motility to 68.23%, reduced ultrastructural abnormalities, elevated acrosomal integrity by 18.7%, and decreased lipid peroxidation by 29% (*P* < 0.05). Proteomic analysis identified 32 differentially expressed proteins. Gene Ontology (GO) enrichment revealed significant involvement in processes related to purine ribonucleoside triphosphate metabolism and transmembrane transporter activity. KEGG pathway analysis indicated predominant enrichment in energy metabolism and signal transduction pathways. PRM validation confirmed that proteins NDUFA8, PGAM2, ACTL7A, PRXL2B, ATP6V0C, and LELP1 exhibited expression patterns consistent with the proteomic data, serving as core biomarkers for skim milk-mediated membrane stabilization.

**Discussion:**

This study provides the first proteomic-level evidence that skim milk enhances the cryotolerance of Inner Mongolia cashmere goat spermatozoa. The mechanism involves the modulation of an energy–membrane protein network, which stabilizes sperm membranes during cryopreservation. The identified proteins establish molecular biomarkers for optimizing semen cryopreservation protocols in this breed.

## 1 Introduction

**FIGURE 1:**
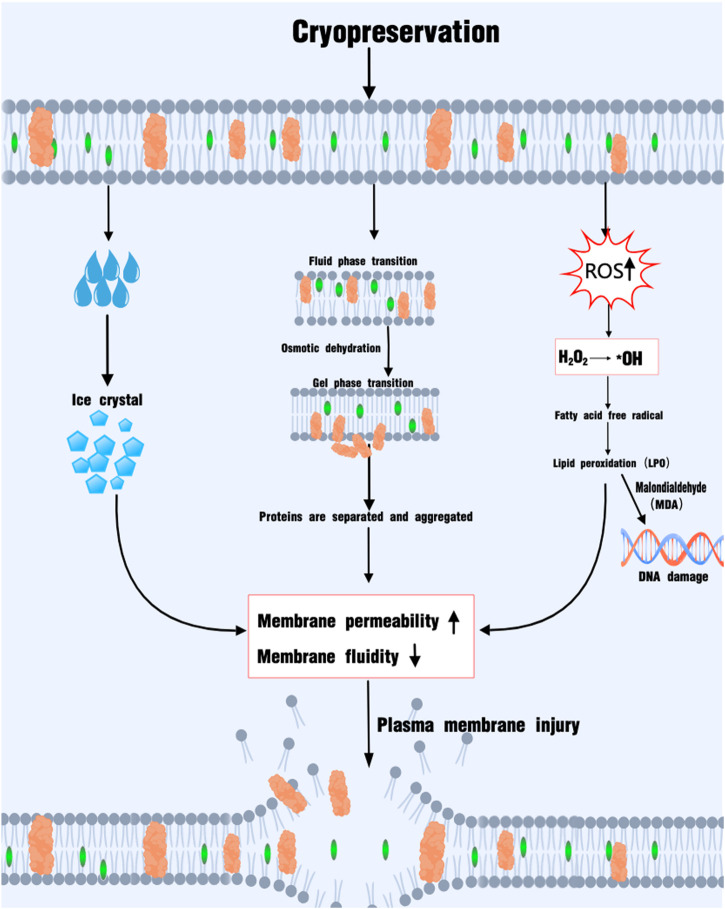
Cryo-damage of the sperm plasma membrane.

The Inner Mongolia cashmere goat (*Capra hircus*), a breed endemic to the cold–arid steppes of northern China, is globally valued for its ultra-fine under-fleece (14–16 µm) and dual-purpose meat production (Abruzzese et al., 2024; Rong et al., 2024). The breed’s genetic uniqueness is underpinned by centuries of natural and artificial selection under conditions of extreme thermal stress, resulting in metabolic adaptations that are reflected at the cellular level—including an unusually high proportion of long-chain polyunsaturated fatty acids (PUFAs, >35% docosahexaenoic acid) in sperm membranes (Agutu et al., 2024). Efficient *ex situ* conservation of elite genetics, therefore, hinges on robust semen cryopreservation protocols. Against this backdrop, semen cryopreservation has emerged as an important technology. It furnishes robust scientific support for use of Inner Mongolia cashmere goat genetic resources, seed-industry innovation, and the cultivation of new-quality productive forces. However, the sperm structure is compromised during cryopreservation, reducing its efficiency (Wang et al., 2021). Sperm cells have less volume and large surface area (Upton et al., 2023), making them sensitive to osmotic pressure changes and ice crystal formation; ice crystals can disrupt the cell membrane, causing mechanical damage (Salehi et al., 2024). Sudden temperature changes can also lead to an increase in reactive oxygen species (ROS), resulting in lipid peroxidation, DNA fragmentation, and mitochondrial dysfunction (Purdy et al., 2013), which can damage the membrane lipid structure and affect sperm motility and fertilization function. The phase transition of sperm membrane lipids and protein denaturation are related to temperature changes (Dhara et al., 2023); during cryopreservation, phospholipids transition undergo a phase from the fluid to gel state, affecting the structure and function of the plasma membrane. The interaction between proteins and phospholipids is weakened, and proteins aggregate into clumps (Zou et al., 2022), disrupting signaling pathways and damaging the plasma membrane (Figure 1).

Cryoprotectants are crucial for maintaining sperm quality and function after freeze–thaw cycles, and optimizing formulations to enhance plasma membrane stability is a key point for technological breakthroughs. Cryoprotectants include permeable and nonpermeable agents, which work synergistically to protect sperm. Permeable cryoprotectants (such as glycerol and ethylene glycol) can penetrate the sperm membrane, replacing bound water to lower the intracellular freezing point and inhibit ice crystal formation, thereby protecting cellular organelles and enzyme systems (Santiani et al., 2017). Non-permeable cryoprotectants (such as yolk and soy lecithin) form a protective film on the surface of sperm, reducing the damage induced by ice crystals to the sperm membrane (Liu et al., 2016). Yolk is rich in nutrition and effectively protects the sperm membrane (Sharma and Sood, 2019), and it is widely used in the cryopreservation of livestock sperm (Oldenhof et al., 2021). Soy lecithin can maintain or enhance sperm viability, motility, and fertilization capacity (Tabarez et al., 2020). Skim milk is an effective cryoprotectant that can enhance sperm freeze resistance and promote recovery of viability (Rodrigues et al., 2020); the lactose in skim milk helps maintain osmotic pressure, milk proteins stabilize the cell membrane, and it also has antioxidant properties, which work better along with permeable cryoprotectants; for example, the combination of glycerol and skim milk can reduce cryoinjury and increase sperm recovery rate and fertilization capacity (McKenna et al., 2020). However, few studies have investigated the combined use of yolk, soy lecithin, and skim milk in sperm diluents and their effects on the sperm membrane.

The study on sperm proteomics is of considerable significance for understanding reproductive processes, fertility regulation, and diagnosis and treatment of related diseases. In recent years, proteomics technology has been widely applied to the study of sperm cryopreservation (Larbi et al., 2024). During cryopreservation, sperm motility and fertilization capacity are reduced due to complex molecular alterations including protein degradation, oxidation, and reorganization (Bogle et al., 2017). Proteomics analysis can facilitate precise identification of proteins associated with cryoinjury, and their expression changes and functional mechanisms can be revealed, facilitating the development of targeted cryoprotective strategies (Peris-Frau et al., 2020). Research has revealed that the decline in sperm motility during cryopreservation is associated with alterations in protein phosphorylation, and through comparative analysis using quantitative phosphoproteomics, proteins such as AKAP3/4 have been identified as potential biomarkers for assessing the quality of frozen–thawed ram sperm (Zang et al., 2024). Additional studies have demonstrated that the distribution pattern of the SPACA1 protein influences sperm fertilization capacity and pregnancy rates following cryopreservation (Minami et al., 2020). In recent years, researchers have conducted comparative proteomics analysis using iTRAQ and found that the PRDX6 protein exerts a protective effect on cryopreserved sperm and that its addition to cryoprotectant solutions can enhance the quality of cryopreserved Mediterranean water buffalo sperm (Luo et al., 2023).

Phosphatidylcholine, a key component in cryopreservation, present in yolk and soy lecithin, can maintain membrane fluidity and reduce ice crystal formation damage by supplementing the components of the sperm plasma membrane phospholipid bilayer. The milk-derived bioactive proteins (such as casein and whey protein) and lactose in skim milk are hypothesized to work synergistically to form a dynamic protective layer on the sperm membrane surface through selective adsorption, potentially stabilizing the sperm plasma membrane structure. Additional studies have used advanced proteomic approaches to identify sperm-specific proteins as fertility markers in ruminants (Larbi et al., 2024). Although previous studies have demonstrated antioxidant properties of whey protein and lactoferrin in various biological systems (Buey et al., 2021; Gad et al., 2011; Rascón-Cruz et al., 2024; Wang et al., 2019), their direct mechanisms in sperm membrane protection during cryopreservation remain to be fully elucidated.

A critical challenge in caprine semen cryopreservation stems from the presence of egg yolk coagulation enzyme (EYCE), also known as phospholipase A, which is naturally secreted by the bulbourethral glands and is present at high concentrations in goat seminal plasma (Roy, 1957). EYCE catalyzes the hydrolysis of phospholipids in egg yolk-based extenders, producing cytotoxic lysophospholipids and free fatty acids that compromise sperm membrane integrity and reduce post-thaw viability (Zou et al., 2022). The detrimental effects of EYCE activity are particularly pronounced in caprine species compared to other ruminants, necessitating modified extender formulations or seminal plasma removal strategies to circumvent enzyme-mediated damage. The replacement or partial substitution of egg yolk with alternative cryoprotectants such as soy lecithin has been explored to minimize EYCE–substrate interactions, although the optimal combination of protective components remains under investigation.

Based on this hypothesis, this study supplemented a yolk–soy lecithin basal extender with skim milk and used tandem mass tag (TMT) quantitative proteomics coupled with parallel reaction monitoring (PRM) validation to characterize the molecular mechanisms underlying skim milk-mediated cryoprotection, providing proteomic-level evidence for optimizing cryopreservation protocols. The specific objectives of this study were to evaluate the effects of varying skim milk concentrations on post-thaw sperm quality parameters in Inner Mongolia cashmere goats, identify differentially expressed membrane proteins associated with cryotolerance enhancement through TMT-based quantitative proteomics, and validate the functional relevance of candidate biomarkers using PRM.

## 2 Materials and methods

### 2.1 Animals

Five sexually mature Inner Mongolia cashmere goat bucks (2 years old, 45 ± 3 kg) in excellent health were selected and housed under identical conditions. Each ram was subjected to a single ejaculation per collection event to minimize potential stress and ensure optimal semen quality.

### 2.2 Experimental design

A cryodiluent based on yolk and soy lecithin was used as the control group. Skim milk at different concentrations (2%, 2.4%, 2.8%, 3.2%, and 3.6%) was added to the basic diluent, and sperm cells were diluted and cryopreserved separately; after thawing, sperm motility and motion parameters were evaluated, the ultrastructural changes in the membranes of frozen–thawed sperm were observed, and plasma membrane integrity, acrosomal integrity, membrane fluidity, and lipid peroxidation levels were analyzed. Subsequently, the plasma membrane was separated, and sperm membrane proteins were extracted. Using quantitative proteomics analysis techniques, proteins with significant differential expression between the experimental and control groups of frozen–thawed sperm were identified, and their functions and regulatory mechanisms were further analyzed.

To minimize individual variation, selected ejaculates from all five breeding rams were pooled in equal proportions at room temperature, yielding approximately 10 mL of pooled semen per collection event. Each pooled semen sample was then divided into equal aliquots and assigned to different experimental groups, including the control group and groups supplemented with varying concentrations of skim milk (2.0%, 2.4%, 2.8%, 3.2%, and 3.6% w/v). The diluted semen was aliquoted into 0.25-mL French cryopreservation straws, with five straws prepared for each treatment group per experimental replicate. Three independent biological replicates were performed for each experimental group, with each replicate representing a distinct semen collection, pooling, processing, and cryopreservation event conducted on separate days. For proteomic analyses (TMT and PRM), three biological replicates per group were analyzed, each derived from an independent cryopreservation experiment.

### 2.3 Sperm collection, cryopreservation, and thawing

Sperm was collected from five healthy 2-year-old Inner Mongolia cashmere goats using an artificial vagina. Sperm density was measured using a sperm densitometer (IMV Technologies, France). Sperm motility was assessed using a computer-assisted sperm analysis system (CASA). Sperm samples with motility exceeding 80% and a density higher than 3 × 10^8^/mL were used for the experiments.

The base diluent used in this experiment consisted of 300 mM Tris, 95 mM citric acid, 55 mM glucose, 15% yolk, 6% glycerol, 1.50% soy lecithin, and 1% antibiotic mixture. According to the experimental design, skim milk at different concentrations: 2%, 2.4%, 2.8%, 3.2%, and 3.6% was added to the base diluent. After preparation, the diluent was filtered using a 0.22-μm filter. The filtered diluent was aliquoted into 50-mL centrifuge tubes and stored at 4 °C. The cryopreservation process began with sperm dilution, followed by gradual cooling of the diluted sperm to a target temperature of 4 °C over a period of 4 h. After the sperm suspension reached 4 °C, an automated semen packaging machine was used to fill the mixture into cryopreservation straws uniformly. After they reached the desired temperature, the sperm cells were packaged into straws and sealed using a sperm packaging machine, then the straws were placed 4 cm above liquid nitrogen, exposed to nitrogen vapor for 7 min, and subsequently stored in liquid nitrogen. After 7 days of storage, the frozen sperm samples were thawed by immersing the straws in a 37 °C water bath for 30 s.

### 2.4 Assessment of sperm motility and kinetic parameters

Sperm total motility (TM) and progressive motility (PM) were assessed using a CASA, along with the following related kinetic parameters: straight-line velocity (VSL), curvilinear velocity (VCL), and average path velocity (VAP). At least five fields of view were examined, with the system capturing at least 100 sperm cells.

### 2.5 Detection of sperm ultrastructure after thawing

The thawed sperm samples were subjected to embedding, followed by trimming, and then sectioned using a LEICA EM UC7 ultramicrotome, obtaining sections 70–90 nm in thickness. The obtained semen sample sections were then mounted on copper grids and stained with 2% uranyl acetate for 15 min, followed by staining with 2% lead citrate for 5 min, ensuring complete immersion of the samples during staining. After staining and drying, the semen sample sections mounted on the copper grids were observed under a transmission electron microscope (TEM; JEM-1200EX, JEOL Ltd., Tokyo, Japan). The embedded semen samples were evenly adhered to carbon conductive tape, sputter-coated with gold for 30 s, and then placed in a scanning electron microscope (SEM; SU-8100, Hitachi Ltd., Tokyo, Japan) for observation.

### 2.6 Detection of sperm plasma membrane integrity

The sperm plasma membrane integrity was assessed using a YO-PRO-1/PI Cell Apoptosis and Necrosis Detection Kit (C1075S, Beyotime Biotechnology, Shanghai, China). The thawed semen was centrifuged at 1,350 r/min for 3 min after adding PBS solution, and the supernatant was removed. This step was repeated twice. The semen was resuspended to a concentration of 1 × 10^6^ cells/mL using 1 × Binding Buffer. An aliquot of 100 μL of sperm suspension was placed in a 1.5 mL centrifuge tube, and 5 μL of YO-PRO-1 was added. The mixture was vortexed and incubated in the dark for 10 min. Then, 5 μL of propidium iodide (PI) dye was added for staining, and the mixture was further incubated in the dark for 5 min. Next, 500 μL of PBS buffer was added, and the mixture was centrifuged at 1,350 r/min three times to remove the supernatant. Finally, 400 μL of 1 × Binding Buffer was added, and the sample was analyzed using a flow cytometer, with an excitation wavelength of 488 nm and emission wavelengths of 530 nm and 630 nm, analyzing approximately 20,000 cells.

Sperm stained with PI were assessed using a confocal laser scanning microscope. PI staining (Ex/Em 535/617 nm) was observed through an emission filter, and images of the sperm were captured using a ×40 objective lens. The image analysis system quantitatively analyzed the intensity of the reaction using a computer. The evaluation process involved the measurement of the integrated optical density (IOD) and mean optical density (MOD) of the sperm.

### 2.7 Detection of sperm acrosome integrity

The sperm acrosomal integrity was detected using a Sperm Acrosome Morphology Peanut Agglutinin Fluorescence Labeling (PNA-FITC) Staining Kit (JM1193, Shanghai Yuduo Biotechnology Co., Ltd., Shanghai, China). After thawing, a certain amount of PBS was added to the semen, which was then centrifuged at 1,350 rpm for 3 min to remove the supernatant. This process was repeated two times, and the sperm were resuspended in PBS to adjust the concentration to 6 × 10^6^ cells/mL. Then, 200 μL of sperm suspension was placed into a 1.5-mL centrifuge tube, and 400 μL of the staining solution was added. The mixture was incubated in the dark at room temperature for 20 min. After incubation, the supernatant was removed by centrifugation, and 400 μL of PI staining solution was added. The mixture was further incubated in the dark at room temperature for 5 min. After removing the supernatant by centrifugation again, the sample was washed twice with 500 μL of PBS. After washing, 50 μL of PBS was added, and the sperm samples were gently resuspended by tapping. Finally, the sample was analyzed using a flow cytometer with an excitation wavelength of 488 nm and emission wavelengths of 530 nm and 630 nm. Approximately 20,000 cells were analyzed to assess the sperm acrosomal integrity.

### 2.8 Detection of sperm membrane fluidity

The sperm membrane fluidity was detected using Merocyanine 540 (GB59294, Glpbio, Montclair, CA, United States) with a YO-PRO-1/PI Cell Apoptosis and Necrosis Detection Kit (C1075S, Beyotime Biotechnology, Shanghai, China). After thawing, sperm cells were stained using Yo-Pro-1 stain at a final concentration of 50 nM and incubated in the dark at room temperature for 12 min. Subsequently, 140 μL of the sperm cell suspension (approximately 6 × 10^6^ cells/mL) was taken, and 30 μL of Merocyanine540 solution (40 μM) was added for further staining, followed by incubation in the dark at room temperature for 10 min. After double staining, the samples were precisely analyzed using a flow cytometer. During detection, the fluorescence signal of the Yo-Pro-1 probe was detected using the FL1 fluorescence channel at a wavelength of 530/30 nm, whereas the fluorescence signal of the Merocyanine 540 probe was detected using the FL2 fluorescence channel at a wavelength of 620 nm. Sperm cells stained with M540 were evaluated using a confocal laser scanning microscope. M540 staining (Ex/Em: 563/607 nm) was observed using an emission filter. Images of the sperm were captured using a ×40 objective lens. The intensity of the reaction was assessed using a computerized image analysis system, measuring the IOD and MOD of the sperm.

### 2.9 Detection of the sperm lipid peroxidation level

The level of sperm lipid peroxidation was detected using the C11-BODIPY581/591 lipid peroxidation fluorescent probe. After thawing, PBS was added to the semen, which was then centrifuged at 1,350 r/min for 3 min to remove the supernatant, and this process was repeated twice, followed by transferring 500 μL of the sperm cell suspension with a concentration of 6 × 106 cells/mL to a tube, and C11-BODIPY581/591 was added to achieve a final concentration of 2 μM for staining, followed by incubation in the dark for 20 min. The samples were analyzed using a flow cytometer with an excitation wavelength of 488 nm and detection at a wavelength of 550 nm. The fluorescence signal of the fluorescent probe was detected using the FITC fluorescence channel, with 10,000 cells detected for each biological replicate in all experiments.

### 2.10 Extraction of sperm membrane proteins from the Inner Mongolia cashmere goat

The thawed semen samples were washed with pre-cooled PBS (500–600 g, 5 min). After removing the supernatant, cells were resuspended in BufferA (200 μL BufferA for initial cell counts less than 6 × 10^6^, and 500 μL BufferA for initial cell counts greater than 6 × 10^6^), incubated on ice for 10 min, and vortexed for 30 s; then, the cell suspension was quickly transferred to a centrifuge tube and centrifuged at 16,000 *g* for 30 s. The centrifuge tube column was discarded, and the precipitate in the receiving tube was resuspended through vigorous vortexing for 10 s, followed by centrifugation at 700 *g* for 2 min; then, the supernatant was transferred to a new centrifuge tube and centrifuged at 4 °C, 16,000 g for 30 min (extended centrifugation time can increase the yield). The supernatant (cytoplasmic component) was discarded, and the precipitate (total membrane component, including organelles and the plasma membrane) was retained and dissolved in 30 µL TEAB.

### 2.11 Proteolysis and TMT labeling

The required samples were transferred to new tubes, and the volume was adjusted to 100 μL using MTEAB. Subsequently, 6 μL of 200 mM MTCEP was added, followed by incubation of the samples at 56 °C for 1 h. An aliquot of 6 μL of 375 mM iodoacetamide was added to the samples, and the samples were incubated in the dark at room temperature for 40 min. Next, 1 μg of trypsin was added, and the samples were digested at 37 °C overnight. An appropriate amount of TMT labeling reagent (TMTpro16plexLabelReagentSet) was removed, followed by equilibration to room temperature. An aliquot of 20 μL of anhydrous acetonitrile was added to the tube and dissolved for 5 min. Then, 20 μL of TMT reagent was added to the digested protein solution, followed by incubation of the reaction mixture at room temperature for 1 h. Each group of samples was combined into three pooled samples, with the control group named A1, A2, and A3 and the skim milk-treated group named B1, B2, and B3. Next, 1 μL of 5% hydroxylamine was added, and the sample was incubated for 15 min to quench the reaction. Finally, the samples were mixed in equal amounts in a new microcentrifuge tube and stored at −80 °C.

### 2.12 Peptide fractionation

The elution solution was prepared for TMT-labeled peptides, with component numbers 1–8 corresponding to ACN concentrations of 5.0%–50.0% and respective volumes, combined with 0.1% TEA. The protective tip at the bottom of the chromatography column was removed, the column was placed in a 2.0-mL sample tube, and centrifuged at 5,000 *g* for 2 min to remove the solution for resin packing, after which the liquid was discarded. An aliquot of 300 μL of ACN was added, followed by centrifugation at 5,000 *g* for 2 min to discard ACN, and this step was repeated once. The column was equilibrated twice with 0.1% TFA solution. The sample was dissolved in 300 μL of 0.1% TFA solution and loaded onto the column, centrifuged at 3,000 *g* for 2 min, and the eluate was retained as the “flow-through” fraction. An aliquot of 300 μL of 5% ACN-0.1% TEA was added and centrifuged at 3,000 *g* for 2 min to remove the unreacted TMT reagent. According to the order of the elution solutions in the table, 300 μL of the appropriate elution solution was added and centrifuged at 3,000 *g* for 2 min to collect fractions. The liquid in each sample tube was evaporated to dryness using a vacuum concentrator.

### 2.13 High-performance liquid chromatography–tandem mass spectrometry (LC-MS/MS)

Chromatographic conditions: trap column: 75 μm i.d.×150 mm, NanoViper C18 3 μm, 100 Å; analytical column: 75 μm i.d.×50 cm, NanoViper C18 2 μm, 100 Å; mobile phase A: 0.1% formic acid; mobile phase B: 0.1% formic acid and 80% ACN. Analysis time per component: 125 min; mass spectrometry conditions: the full scan range of the mass spectrometer is 300–1800 m/z; the resolution of the first-level mass spectrometry is set to 120,000; AGC is standard; maximum IT:AUTO; the resolution of the second-level mass spectrometry is set to 60,000; AGC is custom; maximum IT: custom; NCE/steppedNCE: 36.

### 2.14 Data analysis and identification of differentially expressed proteins (DEPs)

Proteome Discoverer 3.0 software was used to search raw mass spectrometry files. Carbamidomethylation of cysteine was set as a fixed modification, while methionine oxidation and N-terminal acetylation were set as variable modifications. Trypsin was selected as the enzyme, a goat protein database was used, and up to three missed cleavage sites were allowed. Peptide and fragment ion mass tolerances were set to 20 ppm and 0.02 Da, respectively, and protein identification and quantitative analysis were performed. The quantitative information of the protein set was normalized, with the range set to (−1,1). Cluster3.0 software was used to classify protein data based on protein expression levels and sample dimensions. JavaTrewview software was used for visual data analysis, and hierarchical clustering heatmaps were generated to display classification and expression patterns. Differential protein screening was based on fold change (FC) and *P*-value, with criteria of *P*-value <0.05 and FC ≥ 2 (or ≤1/2). Proteins meeting these criteria were considered DEPs, providing a basis for subsequent research.

### 2.15 Functional annotation of DEPs

To explore the potential functions of DEPs, we conducted Gene Ontology (GO) functional annotation and KEGG enrichment analysis. GO annotation was completed using the Quick GO (Gene Ontology Analysis) function of the OmicsBean online tool; KEGG analysis was performed by comparing the target protein sequences with the KEGG Pathway database using Gene Ontology software and conducting KO classification, thereby obtaining the KEGG pathway information in which the DEPs are involved. The DEPs were compared with the GO and KEGG annotation results of all proteins identified and quantified in this experiment, and Fisher’s exact test was used to determine whether the enrichment of proteins in a particular GO term or KEGG pathway is significant, with a *P*-value less than 0.05 as the criterion, thereby identifying the functional categories enriched by all DEPs.

### 2.16 PRM validation of DEPs

Of the 32 DEPs identified by TMT analysis, six proteins were selected for PRM validation based on multiple criteria. Selection was prioritized for proteins exhibiting significant fold-change magnitude (|log_2_FC| > 1.5, P < 0.05) and direct biological relevance to sperm cryotolerance mechanisms, including energy metabolism (NDUFA8 and PGAM2), structural integrity (ACTL7A and LELP1), antioxidant defense (PRXL2B), and membrane homeostasis (ATP6V0C). These proteins also represented major enriched pathways identified in GO and KEGG analyses and possessed reliable proteotypic peptides suitable for PRM quantification. The six selected proteins constitute key functional nodes in the energy–membrane protein network and serve as candidate molecular biomarkers for assessing sperm cryotolerance.

To validate the TMT data, relative quantitative analysis was performed using PRM target proteins. Based on the TMT data, characteristic peptides of target proteins were defined, and only unique peptide sequences were selected for PRM analysis. Prior to the PRM relative quantitative analysis of target proteins, protein preparation and trypsin digestion were performed according to the TMT experimental method. Data for each sample were collected using the PRM acquisition method to quantify the abundance of target proteins. Each group was subjected to three biological replicates. Statistical analysis of data was performed using Prism10 statistical analysis software.

## 3 Results

### 3.1 Effects of skim milk on the post-thaw motility and kinetic parameters of frozen Inner Mongolia cashmere goat sperm

The impact of adding different concentrations of skim milk (2%, 2.4%, 2.8%, 3.2%, and 3.6%) on the cryopreservation of Inner Mongolia cashmere goat semen was evaluated. As shown in Table 1, compared with the control group and other treatment groups, the group in which 2.8% skim milk was added to the diluent showed a significant increase in the post-thaw motility and the proportion of kinetic parameters such as VSL, VCL, and VAP of Inner Mongolia cashmere goat sperm (*P* < 0.05), with the best cryopreservation effect, achieving a post-thaw motility of 68.23% and a progressive motility of 54.67%.

**TABLE 1:** Effects of skimmed milk on motility and motility parameters after freeze–thaw on Inner Mongolia cashmere goat sperm.

Grouping	TM(%)	PM(%)	VAP (μm/s)	VSL (μm/s)	VCL(μm/s)
Control	54.13 ± 3.62^b^	47.37 ± 1.01^b^	47.97 ± 1.93^b^	41.43 ± 1.12^b^	64.20 ± 2.23^b^
2.0%	55.47 ± 2.98^b^	45.40 ± 2.33^b^	48.73 ± 2.95^b^	40.57 ± 2.16^cb^	63.17 ± 1.23^b^
2.4%	55.70 ± 2.01^b^	46.23 ± 2.37^b^	49.33 ± 0.95^b^	38.17 ± 1.69^cd^	62.73 ± 1.97^b^
2.8%	68.23 ± 0.85^a^	54.67 ± 3.92^a^	59.37 ± 1.75^a^	49.33 ± 1.90^a^	77.57 ± 2.69^a^
3.2%	56.67 ± 2.66^b^	38.27 ± 0.47^c^	35.03 ± 4.74^c^	37.30 ± 1.40^d^	63.90 ± 1.15^b^
3.6%	16.37 ± 4.92^c^	25.57 ± 2.00^d^	13.10 ± 1.83^d^	19.70 ± 1.75^e^	37.27 ± 3.16^c^

TM, sperm total motility; PM, progressive motility; VAP, average path velocity; VSL, curvilinear velocity; VCL, straight-line velocity. Values are presented as mean ± standard error of the mean (SEM) from three independent biological replicates. Different superscript letters (a, b, c, d and e) within the same row indicate statistically significant differences among treatment groups (P < 0.05), with groups sharing the same letter not differing significantly (P > 0.05).

### 3.2 Effects of skim milk on the ultrastructure of Inner Mongolia cashmere goat sperm after freeze–thaw

**FIGURE 2:**
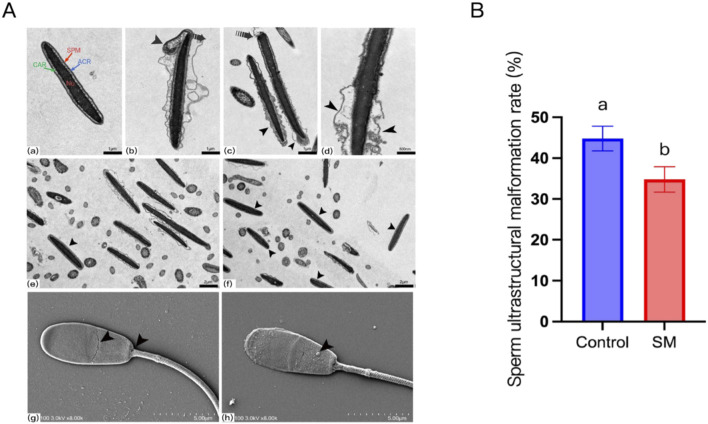
Effects of skim milk on the ultrastructure of Inner Mongolia cashmere goat sperm after freeze–thaw. **(A)** Micrographs of sperm cells from Inner Mongolia cashmere goat semen after freeze-thaw in the control group (b–d, e, g) and the treated group (a, f, h), where (a–f) are transmission electron microscopy (TEM) images and (g,h) are scanning electron microscopy (SEM) images. (Figure a: normal ram sperm with intact plasma and acrosomal membranes and condensed chromatin in the nucleus. spm, sperm plasma membrane; acr, acrosomal membrane; car, nuclear membrane; nu, sperm nucleus. Figures b–d: sperm damaged during the freezing process, resulting in structural changes; Figure b: arrows indicate plasma membrane rupture, separation of the acrosome from the nucleus, head deformation, and irregular swelling of the acrosomal matrix caused by damage induced by freezing; Figures c–d: arrows indicate plasma membrane rupture, head degeneration, and irregular swelling of the acrosomal matrix, while the open arrowheads indicate rupture of the acrosomal membrane and loss of acrosomal contents; Figures e–f: arrows indicate sperm with intact plasma membranes; Figure g: arrows indicate cracks in the sperm membrane and fissures at the junction of the sperm head and neck; Figure h: arrows indicate lipid granule aggregates attached to the sperm surface). **(B)** Ultrastructural analysis of sperm (SM: skim milk-treated group); different letters indicate significant differences (*P* < 0.05).

Electron microscopy revealed significant differences in sperm ultrastructure after freeze–thaw in the 2.8% skim milk treatment group compared with the control group (Figures 2A-e,f). TEM observations revealed that normal ram sperm in the treated group had intact plasma and acrosomal membranes, without any defects or ruptures, smooth surfaces without swelling, and normally shaped nuclei with condensed chromatin (Figure 2A-a). In addition to sperm heads with a normal appearance, malformed sperm cells with altered head plasma membranes and acrosome shapes were identified (Figures 2A-b,d). Figure 2A-b shows the abnormal packaging of sperm chromatin, including vacuoles with myelin-like membranes and granule-containing vacuoles, along with electron-dense inclusions within the sperm chromatin. Figures 2A-c,d show ruptured sperm plasma membranes, deformed heads, and acrosomal matrices characterized by irregular swelling. SEM observations (Figure 2A-g) revealed disordered sperm membranes with cracks or shedding and fissures at the junction of the sperm head and neck. In contrast, the SEM images of sperm from the skim milk-treated group after freeze–thaw showed lipid aggregates attached to the sperm surface (Figure 2A-h), a characteristic that may enhance sperm membrane stability by forming a protective layer to prevent sperm damage during freezing. Microscopic observations clearly demonstrated that skim milk treatment can reduce the incidence of ultrastructural abnormalities in sperm cells after cryopreservation (Figure 2B).

### 3.3 Effects of skim milk on the plasma membrane integrity of Inner Mongolia cashmere goat sperm after freeze–thaw

**FIGURE 3:**
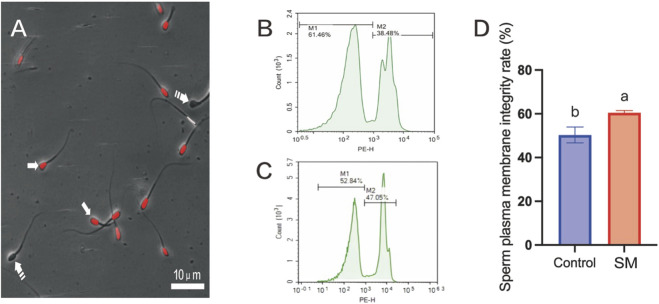
The effect of skim milk on the post-thaw plasma membrane integrity rate of Inner Mongolian cashmere goat sperm. **(A)** Visualization of sperm plasma membrane integrity, with representative microscopic images depicting sperm with damaged plasma membranes. Note: Propidium iodide (PI)-positive sperm cells indicate compromised plasma membrane integrity (arrow), while PI-negative sperm cells indicate intact plasma membranes in live cells (dashed arrow). **(B,C)** Flow cytometry results for plasma membrane integrity. M1, sperm with intact plasma membranes; M2, sperm with damaged plasma membranes. **(D)** Analysis of the sperm plasma membrane integrity rate. Different letters indicate significant differences (P < 0.05).

During the assessment of sperm quality, to precisely determine the integrity of the sperm plasma membrane, PI staining was used in conjunction with flow cytometry; as shown in Figure 3, the plasma membrane integrity of sperm in the 2.8% skim milk treatment group after freeze–thaw was significantly higher than that in the control group (61.46%, *P* < 0.05), indicating enhanced protection of the sperm plasma membrane. Using confocal laser scanning microscopy, fluorescence was observed in the heads of sperm with damaged plasma membranes, while no fluorescence was detected in the heads of sperm with intact membranes.

### 3.4 Effects of skim milk on acrosome integrity of Inner Mongolia cashmere goat sperm after freeze–thaw

**FIGURE 4:**
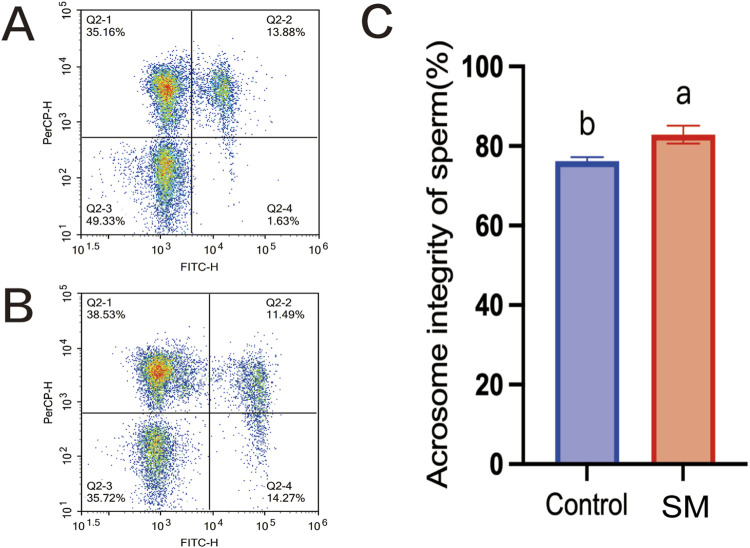
The effect of skim milk on the acrosomal integrity rate of sperm from Inner Mongolian cashmere goats after freeze–thawing. **(A)** Fluorescence density map of the 2.8% skim milk treatment group stained with PNA-FITC/PI. **(B)** Fluorescence density map of the control group stained with PNA-FITC/PI; In the figure, Q2-1: sperm cells with intact acrosomes but damaged plasma membranes; Q2-2: sperm cells with damaged acrosomes and damaged plasma membranes; Q2-3: sperm cells with intact acrosomes and intact plasma membranes; Q2-4: sperm cells with damaged acrosomes but intact plasma membranes. **(C)** Analysis of the sperm acrosomal integrity rate. Different letters indicate significant differences (*P* < 0.05).

To assess the acrosomal integrity rate of sperm, a PNA-FITC/PI staining kit was used, and flow cytometry was used for relevant detection. As shown in Figure 4, the acrosomal integrity rate of sperm after freeze–thawing in the group treated with 2.8% skim milk was significantly higher than that in the control group (84.49%, *P* < 0.05), indicating an enhanced protective effect on the sperm plasma membrane.

### 3.5 The effect of skim milk on the membrane fluidity after freeze–thaw on sperm from Inner Mongolian cashmere goats

**FIGURE 5:**
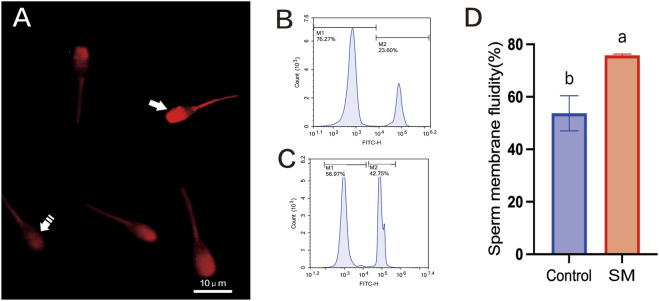
The effect of skim milk on the membrane fluidity after freeze–thaw on sperm from Inner Mongolian cashmere goats. **(A)** Lipid status of the sperm membrane. Fluorescence staining of sperm with stable membranes (M540-negative, indicated by a dashed arrow) and unstable membranes (M540-positive, indicated by an arrow), with increased intensity indicating lipid disorder in the plasma membrane. **(B)** Flow cytometry results for sperm membrane fluidity in the 2.8% skim milk treatment group. **(C)** Flow cytometry results for sperm membrane fluidity in the control group; In the figures, M1: sperm with high membrane fluidity; M2: sperm with low membrane fluidity. **(D)** Analysis of sperm membrane fluidity results. Different letters indicate significant differences (*P* < 0.05).

The Merocyanine 540 fluorescence probe and confocal laser scanning microscopy were used to observe changes in the lipid structure of the sperm cell plasma membrane, with increased fluorescence intensity indicating severe lipid disorder in the plasma membrane (Figure 5A). A Merocyanine 540/Yo-Pro-1 staining kit was used, and flow cytometry was used to assess sperm membrane fluidity (Figures 5B,C). As shown in Figure 5D, the group treated with 2.8% skim milk exhibited a significantly higher sperm membrane fluidity (75.83% ± 0.46%) compared to the control group (53.73% ± 6.68%, *P* < 0.05).

### 3.6 The effect of skim milk on the level of lipid peroxidation after freeze–thaw in sperm from Inner Mongolian cashmere goats

**FIGURE 6:**
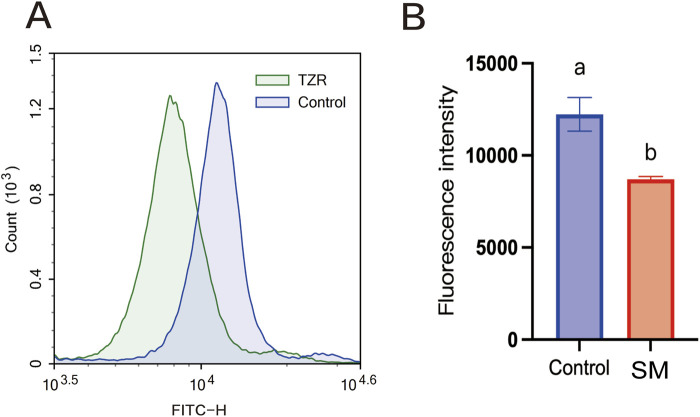
The effect of skim milk on the level of lipid peroxidation after freeze–thaw in sperm from Inner Mongolian cashmere goats. **(A)** Flow cytometry results of lipid peroxidation. **(B)** Statistical analysis of the mean fluorescence intensity of sperm lipid peroxidation. Different letters indicate significant differences (*P* < 0.05).

The level of sperm lipid peroxidation was detected using the C11-BODIPY 581/591 lipid peroxidation fluorescent probe. As shown in Figure 6, the level of sperm lipid peroxidation in the group treated with 2.8% skim milk was lower than that in the control group, with a reduction in the oxidized state, decreased fluorescence intensity in the FITC channel, and a leftward shift of the signal peak (Figure 6A). The average fluorescence intensity of the oxidized state of sperm after freeze–thawing in the skim milk treatment group was significantly lower than that in the control group (Figure 6B) (*P* < 0.05).

### 3.7 Evaluation of protein identification results and cluster analysis

**FIGURE 7:**
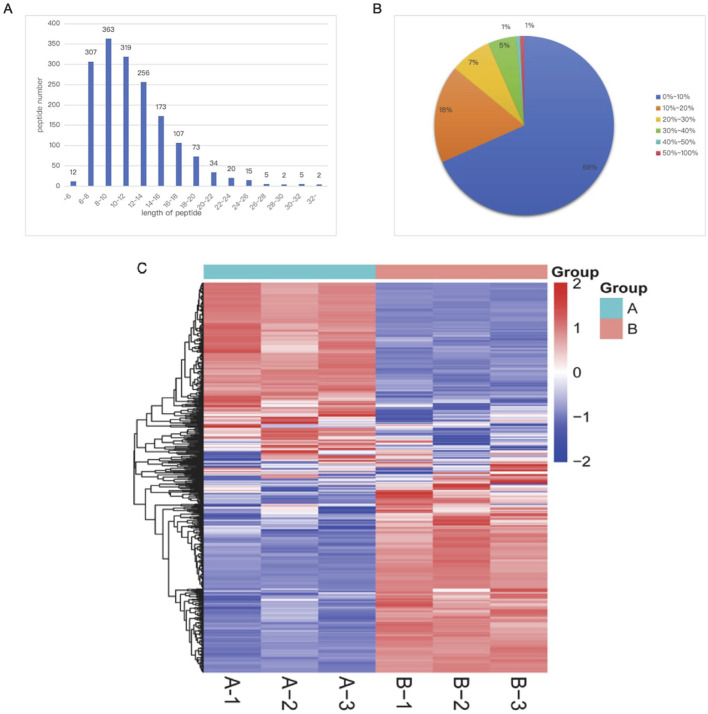
Basic proteomics information of Inner Mongolian cashmere goat sperm. **(A)** Distribution of peptide lengths identified in Inner Mongolian cashmere goat sperm. **(B)** Distribution of protein sequence coverage. **(C)** Protein clustering heatmap. In the figure, **(A,B)** represent the samples of the control group and the skim milk treatment group, respectively. Different colored regions in the figure represent different clustering group information, with the intensity of the color indicating the expression levels of proteins in different samples: red indicates high expression, and blue indicates low expression.

The experiment used a bottom–up proteomics approach to conduct a statistical analysis of the protein expression profile in sperm from Inner Mongolian cashmere goats. A total of 1,693 peptides were identified through spectral analysis. By analyzing these peptides, 549 proteins were successfully identified (Figure 7A). The length and distribution of the identified protein peptides were also statistically analyzed, revealing that the peptide lengths were predominantly distributed between 8 and 22, with most proteins having 8–10 peptides (Figure 7B).

Cluster analysis was performed on the total number of proteins between the 2.8% skim milk treatment group and the control group of frozen semen, as shown in Figure 7C. The results revealed significant differences in the protein expression patterns of sperm samples between the treatment group (Group B) and the control group (Group A), directly reflecting the marked impact of 2.8% skim milk treatment on the protein expression of frozen semen. Further observation revealed that the three samples in Group A (A-1, A-2, and andA-3) and the three samples in Group B (B-1, B -2, B-3) clustered together, respectively, indicating a high degree of similarity in protein expression patterns within the same group.

### 3.8 Differentially expressed protein screening

**FIGURE 8:**
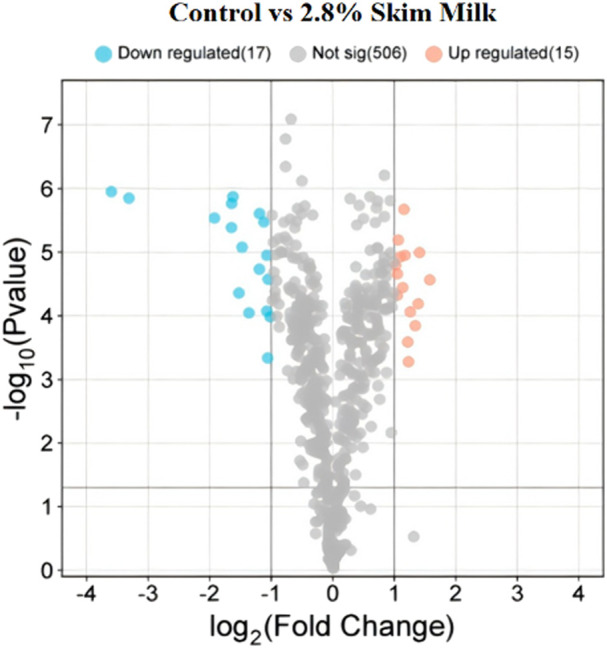
Volcano plot of DEPs between control and 2.8% skim milk treatment groups. The X-axis represents the fold change (logarithmically transformed with base 2), and the Y-axis represents the significance of the difference (P-value) (logarithmically transformed with base 10). Red dots indicate upregulated DEPs in the skim milk treatment group, and blue dots indicate downregulated DEPs. Gray dots represent proteins with no significant change (fold change <2 or *P*-value ≥0.05).

In the process of screening for DEPs, a criterion of *P* < 0.05 and FC ≥ 2 (or ≤1/2) was used. Proteins with FC ≥ 2 were considered upregulated, while those with FC ≤ 1/2 were considered downregulated. A volcano plot was constructed based on the *P*-values and fold changes between the 2.8% skim milk treatment group and the control group (Figure 8), identifying 32 DEPs consisting of 15 upregulated and 17 downregulated proteins. Among the 32 DEPs, 12 were closely related to the regulation of sperm membrane function, namely, NDUFA8, PGAM2, ACTL7A, PRXL2B, ATP6V0C, LELP1, AIMP1, SLC2A3, KNL1, TTLL, VDAC3, and VDAC2 (Table 2).

**TABLE 2:** DEPs related to sperm membrane function.

Protein name	Primary function	Reference
NDUFA8	Provides energy for cells and participates in physiological activities such as cell proliferation and differentiation	Rong et al. (2024)
PGAM2	Involved in sperm energy metabolism, providing energy for sperm motility and function	Guo et al. (2019)
ACTL7A	Involved in the regulation of sperm-zona pellucida fusion	Yamatoya et al. (2020)
PRXL2B	Involved in the redox regulation of sperm cells	Agnieszka et al. (2025)
ATP6V0C	Involved in membrane trafficking of cells	Mattison et al. (2023)
LELP1	Aberrant expression may affect the normal generation of sperm	Chen et al. (2023)
AIMP1	Involved in the assembly of cytoskeletal protein complexes	Jackson et al. (2011)
SLC2A3	Also known as GLUT3, it is involved in sperm energy metabolism and supports sperm maturation	Kishimoto et al. (2015)
KNL1	Involved in the assembly and regulation of spindle fibers during cell division	Zhao et al. (2023)
TTLL	Involved in the regulation of tubulin glutamylation, indirectly affecting the distribution and function of sperm membrane proteins	Chawla et al. (2016)
VDAC3	Abnormalities in the structure and function of VDAC3 are associated with reduced sperm motility	Asmarinah et al. (2012)
VDAC2	Involved in the regulation of energy and metabolic exchange between sperm mitochondria and cytoplasm	Conti Nibali et al. (2024)

### 3.9 Functional enrichment analysis of DEPs

**FIGURE 9:**
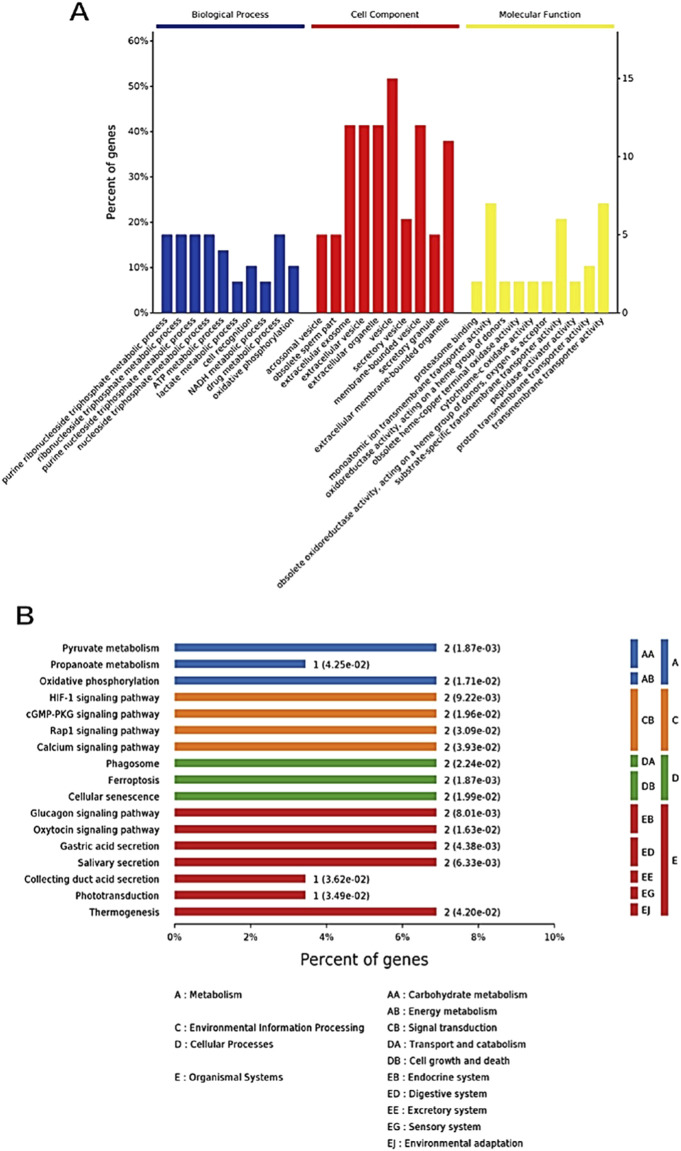
Functional enrichment analysis of DEPs. **(A)** GO enrichment analysis statistical graph (top 10 entries); **(B)** KEGG significantly enriched the pathway annotation map.

The GO results showed that the DEPs were significantly enriched in 10 biological processes (BPs), 10 molecular functions (MFs), and 10 cellular components (CCs), where the BP terms included the metabolic processes of purine ribonucleoside triphosphate, ribonucleoside triphosphate, and purine nucleotide triphosphate; the CC terms included extracellular exosomes, extracellular vesicles, extracellular organelles, vesicles, and membrane-bound vesicles; and the MF terms were most significantly enriched in single-atom ion transmembrane transporter activity, specific transmembrane transporter activity, and transmembrane transporter activity (Figure 9A).

Through KEGG enrichment analysis of the DEPs, we found that these proteins were mapped to 25 different metabolic pathways. Based on the set significance threshold, 17 of these pathways showed significant enrichment (Figure 9B; Supplementary Figures S1-S17). These pathways cover a variety of biological processes and metabolic activities, particularly including: pyruvate metabolism, propanoate metabolism, oxidative phosphorylation, HIF-1 signaling pathway, cGMP–PKG signaling pathway, Rap1 signaling pathway (Rap1), calcium signaling pathway, phagosome, ferroptosis, cellular senescence, glucagon signaling pathway, oxytocin signaling pathway, gastric acid secretion, salivary secretion, collecting duct acid secretion, phototransduction, and thermogenesis.

### 3.10 PRM validation of protein expression

**FIGURE 10:**
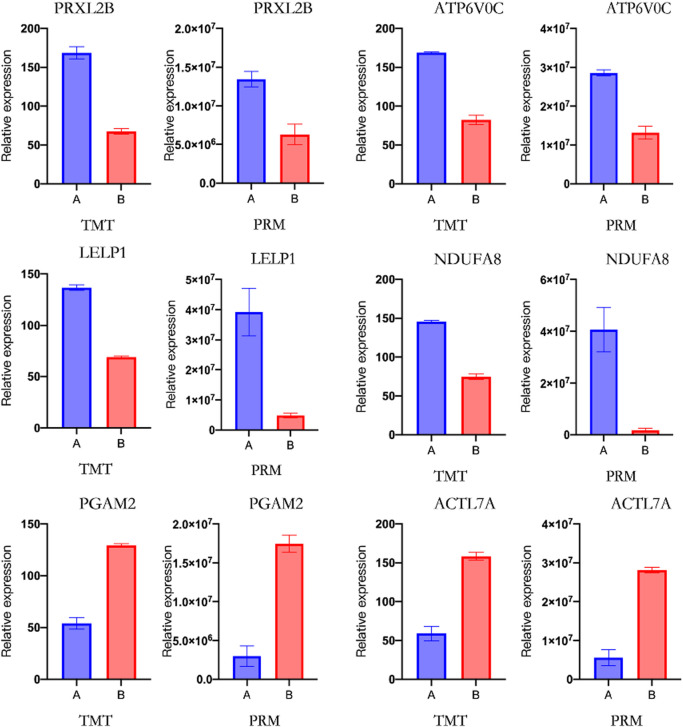
PRM validation of DEPs. The bar graphs show relative abundance of six selected proteins (NDUFA8, PGAM2, ACTL7A, PRXL2B, ATP6V0C, and LELP1) in the control group and 2.8% skim milk treatment group. Data are presented as mean ± SEM from three biological replicates. Asterisks indicate significant differences between groups (*P < 0.05 and **P < 0.01). The PRM results confirm the expression patterns observed in TMT proteomics analysis, validating these proteins as key regulators of sperm membrane stability during cryopreservation.

PRM technology has high specificity and sensitivity in the quantitative analysis of target protein expression levels. In this study, we used PRM technology for quantitative analysis of 12 DEPs related to sperm membrane function. The results are shown in Figure 10, where six key proteins showed consistency between TMT and PRM analysis results, including four upregulated proteins (PRXL2B, ATP6V0C, LELP1, and NDUFA8) and two downregulated proteins (PGAM2 and ACTL7A). This result fully confirms the high reliability and reproducibility of TMT quantitative data.

**FIGURE 11:**
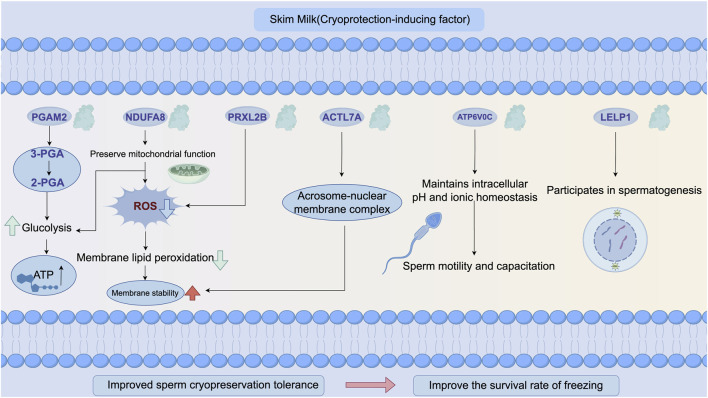
A roadmap for the synergistic mechanism of skim milk in enhancing sperm cryoresistance.

As illustrated in Figure 11, the six validated DEPs form a coordinated energy–membrane protein network that underlies the cryoprotective effects of skim milk supplementation. This network integrates the six PRM-validated DEPs with their functional roles and phenotypic outcomes. Upregulated proteins (PGAM2, ACTL7A, NDUFA8, and LELP1) are involved in energy metabolism, structural integrity, and membrane stability, while downregulated proteins (PRXL2B and ATP6V0C) are associated with antioxidant defense and membrane trafficking. The coordinated modulation of these proteins correlates with improved post-thaw quality: enhanced motility (68.23% vs. 52.15%, *P* < 0.01), increased acrosomal integrity (+18.7%, *P* < 0.05), and reduced lipid peroxidation (−29%, *P* < 0.05). Skim milk components (lactoferrin, whey proteins, and lactose) are proposed to stabilize sperm membranes and provide exogenous antioxidant capacity, triggering adaptive proteomic responses that optimize energy metabolism and maintain structural integrity. All six proteins were validated by PRM with high concordance to TMT quantification (r > 0.85), and NDUFA8 expression positively correlated with post-thaw motility (r = 0.78, *P* < 0.01).

## 4 Discussion

Sperm cryopreservation technology has remarkable advantages in the field of animal reproduction, enabling long-term storage of semen and thereby considerably enhancing the utilization rate of superior rams, providing an important safeguard for the efficient use of livestock genetic resources. However, the cryopreservation and thawing process can damage the sperm membrane, which, as a vital component of sperm structure, determines its stability in changing environments and directly affects its fertilization capability (Ozimic et al., 2023). The damage to the sperm membrane during cryopreservation mainly manifests as disruption of membrane structure, alteration in lipid composition, and functional decline, all of which can reduce sperm motility (Chakrabarty et al., 2007). To address the aforementioned issues, the research field generally focuses on optimizing formulations, enhancing sperm cryoresistance, and reducing cryopreservation-induced damage as core directions for improvement. Yolk and soy lecithin have complementary advantages in protecting sperm, and when used in combination, they can fully leverage their respective strengths to synergistically enhance sperm survival during the freeze–thaw processes. This approach optimizes the limitations of single-component cryoprotectants, providing more comprehensive protection for sperm cells during the freeze–thaw process (Salmani et al., 2014; Sieme et al., 2016). The casein and whey proteins in skim milk can form a dynamic protective layer on the sperm membrane surface through selective adsorption, stabilizing the sperm plasma membrane structure. Therefore, we explored the effects of adding different concentrations of skim milk to a cryodiluent based on yolk–soy lecithin on the frozen sperm of Inner Mongolian cashmere goats. The results showed that the addition of 2.8% skim milk could markedly enhance sperm motility and movement parameters after freeze–thawing when combined with other non-permeable cryoprotectants, thereby alleviating the impact of freeze–thaw damage on sperm.

During sperm cryopreservation, mechanical damage from intracellular ice crystals and osmotic imbalance caused by the low-temperature environment can lead to lipid phase transitions in the sperm cell membrane, inducing plasma membrane rupture and acrosomal vesicle leakage (Kalthur et al., 2008; Shi et al., 2024). Therefore, we combined scanning electron microscopy, transmission electron microscopy, and flow cytometry for analysis and found that treatment with 2.8% skim milk reduced the incidence of morphological abnormalities in sperm after freeze–thawing while enhancing plasma membrane and acrosomal integrity, indicating that, to some extent, synergistic effects between different diluents enhanced membrane stability. This may be related to components such as whey proteins in skim milk.

The beneficial effects of skim milk supplementation may be partially attributed to its potential modulatory effects on EYCE activity. Goat seminal plasma contains high levels of EYCE, a phospholipase that hydrolyzes egg yolk phospholipids to produce cytotoxic lysophospholipids and free fatty acids, thereby compromising sperm membrane integrity during cryopreservation (Roy, 1957; Zou et al., 2022). The optimal skim milk concentration identified in this study (2.8%) may represent a balance between providing sufficient protein-mediated enzyme inhibition and maintaining appropriate osmotic conditions for sperm survival.

Previous studies have confirmed that supplementation with whey proteins can considerably improve sperm quality in mice (Ketheeswaran et al., 2020). The hydrophobic residues of whey proteins can interact with the hydrophobic tails of the phospholipid bilayer, thereby stably anchoring to the membrane (Permyakov, 2020). Thus, whey proteins in skim milk may bind to the phospholipid bilayer in the sperm membrane, optimize the membrane lipid composition ratio, and form a stable protective layer that effectively resists the impact of ice crystals and osmotic pressure changes on the sperm membrane during cryopreservation.

Lipid peroxidation (LPO) is a key factor affecting sperm functional defects in cryopreserved semen. It directly causes damage to PUFAs in the sperm membrane, making sperm more susceptible to environmental stress. In this study, the addition of 2.8% skim milk enhanced sperm membrane fluidity and reduced LPO levels after freeze–thawing. Studies have shown that LPO can damage sperm membrane properties, reduce membrane fluidity, and impair fertilization capacity (Aitken, 2017). Other studies have indicated that the freezing process directly depletes endogenous sperm antioxidant levels and promotes the cascade production of ROS through mitochondrial uncoupling, creating a vicious cycle of oxidative damage and antioxidant depletion (Ponchia et al., 2021; Shi et al., 2024). The protective effect observed may be related to the antioxidant properties of lactoferrin in skim milk. One study confirmed that lactoferrin can significantly enhance total sperm antioxidant capacity and alleviate sperm damage by activating the Nrf2/ARE signaling pathway (Li et al., 2023). This finding not only reveals the potential of skim milk as a cryoprotectant in reducing sperm oxidative stress but also further highlights its important role in maintaining sperm function.

The sperm membrane structure plays a crucial role in the regulation of sperm motility, acrosome reaction, fertilization, and other processes, with membrane proteins, as key functional components of the sperm membrane structure, participating in many important physiological activities such as signal transduction, host defense, and maintenance of cellular homeostasis (Tosaka and Kamiya, 2023). Numerous studies have shown that there is a close relationship between cryodamage and changes in the expression levels of sperm proteins (Lv et al., 2020; Macleod and Varmuza, 2013). Therefore, this study extracted sperm membrane proteins from the control group and the 2.8% skim milk treatment group after thawing, and utilized TMT labeling-based quantitative proteomics to systematically analyze the regulatory effects of skim milk on sperm membrane stability. The study results revealed significant differences in the expression patterns of total proteins between the skim milk treatment group and the control group of frozen semen, indicating that skim milk not only enhances the sperm survival rate and functional integrity after thawing but also potentially maintains physiological functions of sperm after thawing by regulating the expressions of membrane proteins. The results of differential protein identification showed that 32 DEPs were identified between the control group and the skim milk treatment group, consisting of 15 upregulated proteins and 17 downregulated proteins, among which proteins closely related to the regulation of sperm membrane function include NDUFA8, PGAM2, ACTL7A, PRXL2B, ATP6V0C, LELP1, AIMP1, SLC2A3, KNL1, TTLL, VDAC3, and VDAC2. GO enrichment analysis revealed that among proteins closely related to sperm membrane function, DEPs were significantly enriched in GO terms associated with biological processes and molecular functions, such as the metabolism of purine ribonucleoside triphosphates (NTPs) and transmembrane transporter activity. KEGG enrichment analysis showed that DEPs were significantly enriched in pathways related to energy metabolism and signal transduction.

This study conducted PRM validation of DEPs related to sperm membrane function, and the results indicate that the expression changes in upregulated proteins PRXL2B, ATP6V0C, LELP1, and NDUFA8 and downregulated proteins PGAM2 and ACTL7A were consistent with the trends observed in TMT proteomics analysis. These results not only validated the reliability of TMT labeling-based quantitative proteomics analysis but also further revealed the potential roles of the aforementioned DEPs in the regulation of sperm membrane function. In previous studies, LELP1 was found to be associated with spermatogenesis (Chen et al., 2023), but the regulatory role of LELP1 in stabilizing sperm membranes by skim milk remains unclear. Glycolysis is an important pathway for sperm energy metabolism, and the efficient operation of the glycolytic pathway is crucial for maintaining the fluidity and function of the sperm membrane (Abruzzese et al., 2024; Mateo-Otero et al., 2023). Phosphoglycerate mutase 2 (PGAM2) is a key enzyme in the glycolytic pathway, which catalyzes the conversion of 3-phosphoglycerate to 2-phosphoglycerate (Yang et al., 2016), a key step in ATP production during glycolysis (Takei et al., 2014). PRM validation showed high expressions of PGAM2 in the skim milk treatment group possibly because during the cryopreservation of Inner Mongolian cashmere goat semen, skim milk upregulated it to enhance glycolytic activity and provide ATP for sperm motility. Studies have found that PGAM2, a protein related to energy metabolism, is abnormally expressed in patients with asthenozoospermia (Guo et al., 2019), further confirming the important role of PGAM2 in sperm motility regulation. Ubiquinone oxidoreductase (NDUFA8) is an important component of mitochondrial respiratory chain complex I, playing an important role in cellular energy metabolism and function (Fu et al., 2024). Enhanced activity of mitochondrial respiratory chain complex I promotes efficient ATP production, which is beneficial for sperm motility and fertilization (Vahedi Raad et al., 2024). In this study, upregulation of NDUFA8 expression in the skim milk treatment group represents adaptive compensation for cryopreservation-induced mitochondrial dysfunction. Enhanced NDUFA8 levels likely restore electron flow efficiency and ATP production essential for flagellar motility recovery. Studies have shown that complex I dysfunction is a primary cause of reduced sperm motility after freeze–thaw (Vahedi Raad et al., 2024). The coordinated upregulation of NDUFA8 (oxidative phosphorylation) and PGAM2 (glycolysis) represents a dual-pathway energy optimization strategy ensuring ATP availability throughout the flagellum. Importantly, this suggests that skim milk supplementation preserves mitochondrial functional integrity, a critical determinant of fertilization competence, by supporting both glycolytic and mitochondrial energy pathways.

The proteins whose expression trends were consistent between PRM validation and TMT proteomics analysis also included proteins related to membrane structure integrity and signal transduction, such as testis-specific actin-like 7A (ACTL7A), peroxiredoxin-like 2B (PRXL2B), and ATPase H+ transporting V0 subunit c (ATP6V0C). ACTL7A protein is a testis-specific actin that forms physical cross-links with the F-actin network on the inner side of the acrosomal membrane through its C-terminal domain, which may enhance the mechanical stability of the acrosome–nuclear membrane complex and prevent membrane rupture caused by ice crystal formation during cryopreservation (Ferrer et al., 2023). Studies have found that the absence of ACTL7A leads to the separation of the acrosome from the nucleus, causing sperm deformity (Zhou et al., 2023). The results of this study suggest that skim milk may upregulate ACTL7A expression to stabilize the acrosome–nuclear membrane complex structure and protect the integrity of sperm ultrastructure. In addition, α-lactalbumin and β-lactoglobulin in skim milk may be embedded into the lipid raft microdomains of the sperm membrane through hydrophobic interactions, and their amphipathic structures can both decrease the phase transition temperature of membrane lipids during cryopreservation and stabilize the transmembrane helical conformation of membrane proteins (Darmawan et al., 2020). This auxiliary anchoring effect of exogenous proteins may form a synergistic effect, with the endogenous cytoskeletal support mediated by ACTL7A to jointly maintain the stability of the membrane structure.

PRXL2B is a protein related to peroxiredoxins, which typically has the function of scavenging ROS and regulating the cellular antioxidant defense mechanism (Averill-Bates, 2024). The results of this study found that skim milk significantly downregulated the expression of PRXL2B, and no relevant literature has been found to prove the possible regulatory role of its downregulation, but proteins are prone to denaturation, aggregation, or degradation during cryopreservation, reducing the stability of the PRXL2B protein and downregulating its expression. Another possibility is that skim milk, as a cryoprotectant, reduces cryodamage to some extent, but its components or protective capacity are limited and fail to adequately protect key antioxidant proteins such as PRXL2B, leading to their easy inactivation and degradation. However, these explanations do not adequately account for the paradox that lipid peroxidation decreased by 29% (P < 0.05), despite PRXL2B downregulation. An alternative mechanism may involve compensatory antioxidant activity from skim milk components, particularly lactoferrin and whey proteins with documented ROS-scavenging properties (Gad et al., 2011; Rascón-Cruz et al., 2024), which reduce cellular dependence on endogenous PRXL2B expression. Enhanced membrane integrity may also prevent initial lipid peroxidation events, thereby decreasing ROS generation at the source. Further validation through direct ROS measurements and component-specific depletion experiments is warranted.

ATP6V0C is an important subunit of V-ATPase, mainly involved in proton transport, maintaining intracellular pH and ion balance, and is crucial for sperm survival, motility, and capacitation (Mangieri et al., 2014; Pamarthy et al., 2018; Santos-Pereira et al., 2021). In this study, the expression of ATP6V0C in sperm after thawing following skim milk treatment showed a downward trend, which may be due to decreased membrane lipid fluidity and the denaturation or degradation of membrane proteins caused by cryopreservation and thawing, thereby reducing ATP6V0C synthesis or protein stability.

Several limitations should be acknowledged. This study evaluated skim milk exclusively within a yolk-soy lecithin basal extender without direct comparisons to commercial extenders, which would better contextualize practical implementation. Of 32 DEPs identified, only six were validated by PRM, although strategically prioritized based on biological relevance. The proposed mechanisms of whey protein and lactoferrin cryoprotection were inferred from the literature rather than directly validated in our experimental design. Cross-species validation in other ruminants with different membrane compositions would establish the generalizability. Despite these limitations, our study provides the first proteomic characterization of skim milk cryoprotection in caprine spermatozoa.

## 5 Conclusion

This study comprehensively investigates the protective effects of skim milk on sperm membrane stability during cryopreservation of Inner Mongolian cashmere goat semen and elucidates the underlying mechanisms through proteomic analysis. The study demonstrates that adding 2.8% skim milk to a yolk–soy lecithin-based extender significantly enhances the post-thaw quality of Inner Mongolian cashmere goat sperm by improving membrane stability and reducing cryodamage. The optimized formulation resulted in a significant increase in sperm motility to 68.23%, an 18.7% improvement in acrosomal integrity, enhanced membrane fluidity, and a 29% reduction in ultrastructural abnormalities and lipid peroxidation levels. By integrating TMT-based proteomics and PRM validation, we identified 32 DEPs, with six—NDUFA8, PGAM2, ACTL7A, PRXL2B, ATP6V0C, and LELP1—confirmed as core markers regulating membrane stability. Functional enrichment analysis revealed that these proteins play crucial roles in energy metabolism (e.g., oxidative phosphorylation and glycolysis) and transmembrane transport processes. These findings provide the first proteomic-level evidence that skim milk exerts its cryoprotective effects through a coordinated energy–membrane protein network, stabilizing sperm membranes by enhancing antioxidant capacity, metabolic support, and structural integrity. This study provides new theoretical and practical insights for optimizing cryopreservation techniques for cashmere goats and other livestock, offering a significant application value for improving the utilization efficiency of superior ram genetic resources and accelerating breed improvement.

## Data Availability

The datasets presented in this study can be found in online repositories. The names of the repository/repositories and accession number(s) can be found in the article/Supplementary Material.
