# Supplementary material for: Exploring the effect of skim milk on the membrane stability of frozen–thawed Inner Mongolia cashmere goat sperm based on proteomics
Source: Front Cell Dev Biol. 2026 Jan 14;13:1701830. doi: 10.3389/fcell.2025.1701830 (PMC12846951; doi:10.3389/fcell.2025.1701830)
Supplement: Supplementary file 1 [file DataSheet1.docx]

# Appendix

Appendix 1 List of Significantly Differentially Expressed Proteins

| Accession | Gene Name | average A | average B | FC(A vs B) | P_Value |
| --- | --- | --- | --- | --- | --- |
| A0A452F8F4 | AIMP1 | 0.003197453 | 0.001071035 | 2.9853857 | 0.0000273 |
| A0A452FWR3 | KNL1 | 0.002546153 | 0.00095749 | 2.6591950 | 0.0000102 |
| A0A452EFC3 | PRXL2B | 0.003155449 | 0.001204545 | 2.6196180 | 0.0000650 |
| A0A452DZE4 | LOC102175985 | 0.003640265 | 0.001435891 | 2.5351964 | 0.0001437 |
| A0A452EWE7 | COX5B | 0.002879666 | 0.001201678 | 2.3963717 | 0.0000870 |
| A0A452FFW1 | VDAC3 | 0.003297964 | 0.001403889 | 2.3491624 | 0.0005287 |
| A0A452DUV6 | SMCP | 0.002674266 | 0.001148114 | 2.3292684 | 0.0002590 |
| A0A452F873 | COX6B2 | 0.002727706 | 0.001207993 | 2.2580474 | 0.0000112 |
| A0A452EEN1 | EFHB | 0.002524253 | 0.001130985 | 2.2319068 | 0.0000021 |
| A0A452EGD9 | VDAC2 | 0.00303951 | 0.001381322 | 2.2004359 | 0.0000361 |
| A0A452FRS6 | ATP6V0C | 0.003158639 | 0.00146916 | 2.1499617 | 0.0000118 |
| A0A452EJG9 | SPMIP6 | 0.002884833 | 0.001378488 | 2.0927520 | 0.0000065 |
| A0A452F4M2 | LDH | 0.002690406 | 0.001298243 | 2.0723441 | 0.0000477 |
| A0A452DMD1 | LELP1 | 0.002556753 | 0.001234124 | 2.0717155 | 0.0000220 |
| A0A452FT17 | NDUFA8 | 0.002720955 | 0.001337344 | 2.0345954 | 0.0000161 |
| A0A452F2I4 |  | 0.00123073 | 0.002490978 | 0.4940748 | 0.0001040 |
| A0A452EXX7 | LOC108636372 | 0.001191886 | 0.002464172 | 0.4836863 | 0.0000267 |
| A0A452FLX5 | LOC102188781 | 0.000543811 | 0.001129153 | 0.4816099 | 0.0004615 |
| A0A452FQ64 | HAGH | 0.001347952 | 0.00282828 | 0.4765979 | 0.0000112 |
| A0A452FJG5 | PKD2L2 | 0.001105926 | 0.002322791 | 0.4761196 | 0.0000850 |
| A0A452EAT2 | ACE | 0.001145841 | 0.002485929 | 0.4609307 | 0.0000034 |
| A0A452EME5 | NME5 | 0.00093221 | 0.002125904 | 0.4385006 | 0.0000025 |
| A0A452DR91 | PGAM2 | 0.001010168 | 0.002307205 | 0.4378318 | 0.0000186 |
| A0A452DNE7 | ACTL7A | 0.001100689 | 0.002826264 | 0.3894501 | 0.0000906 |
| A0A452FCU6 | EDIL3 | 0.000873065 | 0.002423098 | 0.3603096 | 0.0000084 |
| A0A452E1I4 | ADAM2 | 0.000898324 | 0.002583033 | 0.3477787 | 0.0000440 |
| A0A452FVI4 | TTLL2 | 0.001209118 | 0.003719004 | 0.3251187 | 0.0000013 |
| A0A452FBL5 | LCORL | 0.001088416 | 0.003398305 | 0.3202820 | 0.0000017 |
| A0A452F4B6 | VCP | 0.00090468 | 0.002830327 | 0.3196378 | 0.0000041 |
| A0A452EB21 | SLC2A3 | 0.000712785 | 0.002701716 | 0.2638268 | 0.0000029 |
| A0A452FQ74 | PSME4 | 0.000464661 | 0.004613998 | 0.1007067 | 0.0000014 |
| A0A452EW27 | LOC102172488 | 0.000388062 | 0.004699671 | 0.0825722 | 0.0000011 |
| A0A452F8F4 | AIMP1 | 0.003197453 | 0.001071035 | 2.9853857 | 0.0000273 |

Appendix 2 Enrichment Analysis of the KEGG Pathway for Differentially Expressed Proteins

| Pathway ID | Pathway Name | Count | Pop Hit | List_Total | Pvalue |
| --- | --- | --- | --- | --- | --- |
| oas00620 | Pyruvate metabolism | 2 | 50 | 12 | 0.00187 |
| oas04216 | Ferroptosis | 2 | 50 | 12 | 0.00187 |
| oas04971 | Gastric acid secretion | 2 | 77 | 12 | 0.00438 |
| oas05012 | Parkinson disease | 3 | 291 | 12 | 0.00568 |
| oas04970 | Salivary secretion | 2 | 93 | 12 | 0.00633 |
| oas04922 | Glucagon signaling pathway | 2 | 105 | 12 | 0.00801 |
| oas04066 | HIF-1 signaling pathway | 2 | 113 | 12 | 0.00922 |
| oas05010 | Alzheimer disease | 3 | 410 | 12 | 0.0146 |
| oas05418 | Fluid shear stress and atherosclerosis | 2 | 147 | 12 | 0.0153 |
| oas04921 | Oxytocin signaling pathway | 2 | 152 | 12 | 0.0163 |
| oas00190 | Oxidative phosphorylation | 2 | 156 | 12 | 0.0171 |
| oas04022 | cGMP-PKG signaling pathway | 2 | 168 | 12 | 0.0196 |
| oas04218 | Cellular senescence | 2 | 169 | 12 | 0.0199 |
| oas04145 | Phagosome | 2 | 180 | 12 | 0.0224 |
| oas05022 | unkown | 3 | 499 | 12 | 0.0246 |
| oas05152 | Tuberculosis | 2 | 211 | 12 | 0.0301 |
| oas04015 | Rap1 signaling pathway | 2 | 214 | 12 | 0.0309 |
| oas04613 | unkown | 2 | 227 | 12 | 0.0344 |
| oas05415 | unkown | 2 | 228 | 12 | 0.0347 |
| oas04744 | Phototransduction | 1 | 27 | 12 | 0.0349 |
| oas04966 | Collecting duct acid secretion | 1 | 28 | 12 | 0.0362 |
| oas04020 | Calcium signaling pathway | 2 | 244 | 12 | 0.0393 |
| oas05208 | unkown | 2 | 252 | 12 | 0.0417 |
| oas04714 | Thermogenesis | 2 | 253 | 12 | 0.042 |
| oas00640 | Propanoate metabolism | 1 | 33 | 12 | 0.0425 |

Appendix 3 Results of Relative Quantitative Analysis of Target Protein PRM

| Protein ID number | Peptide sequence | Protein name | The mean value of protein quantification for each group of samples in PRM | |
| --- | --- | --- | --- | --- |
|  |  |  | A | B |
| A0A452EFC3 | WIARDLSNLK | PRXL2B | 13464166 | 6275800 |
| A0A452FRS6 | GTAQQPR | ATP6V0C | 28595033 | 13172233 |
| A0A452DMD1 | QCEPGCEQK | LELP1 | 39212666 | 4808233 |
| A0A452FT17 | ARPEPNPEVEGDLKPAR | NDUFA8 | 40632333 | 1793966 |
| A0A452DR91 | FLGDEETVR | PGAM2 | 2985133 | 17461133 |
| A0A452DNE7 | ETFVGHELINPEVR | ACTL7A | 5594033 | 28144453 |


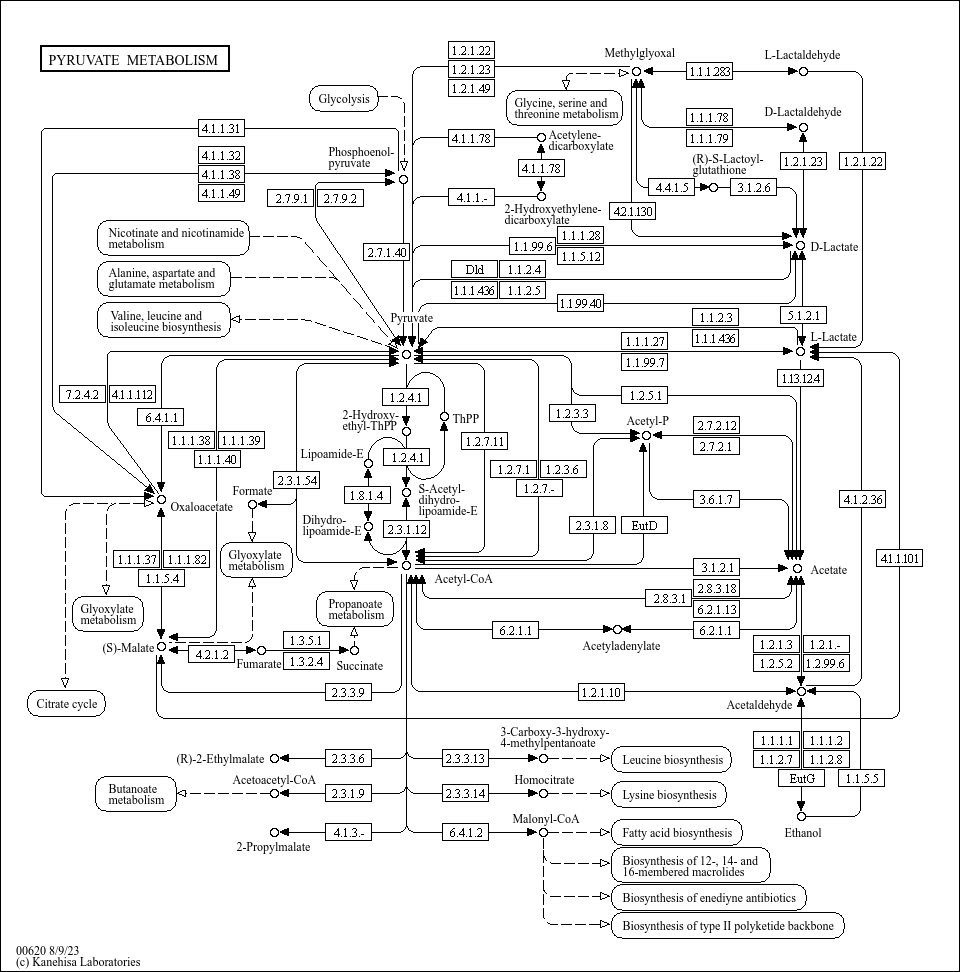

Figure 1: Pyruvate Metabolic Pathway


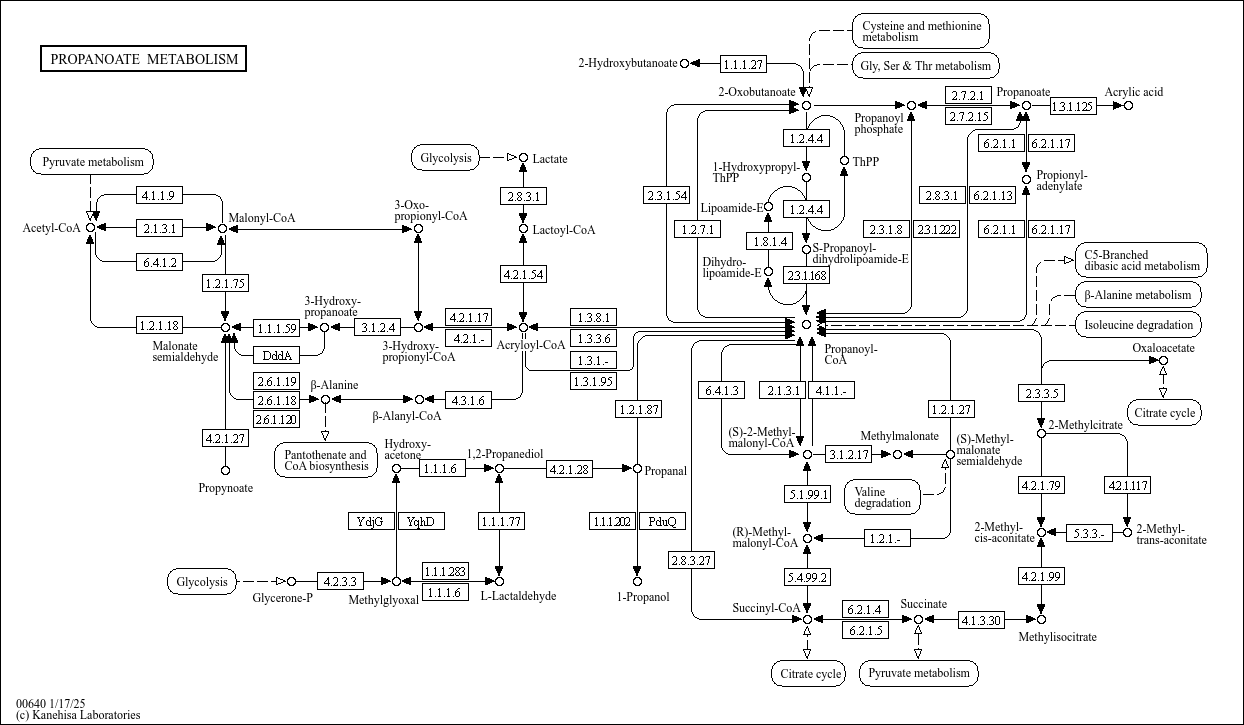


Figure 2: Propionic Acid Metabolic Pathway


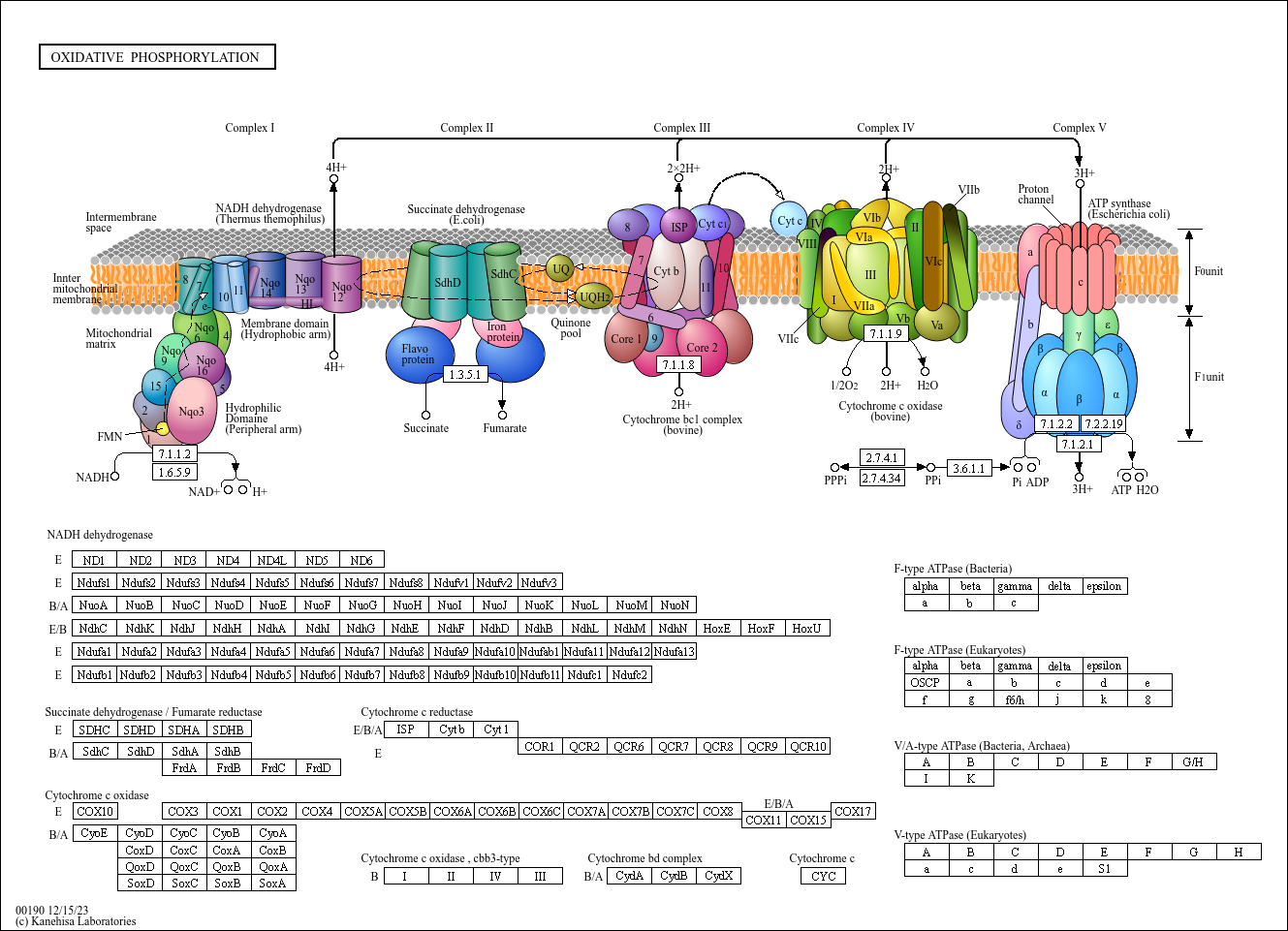


Figure 3: Oxidative Phosphorylation Pathway
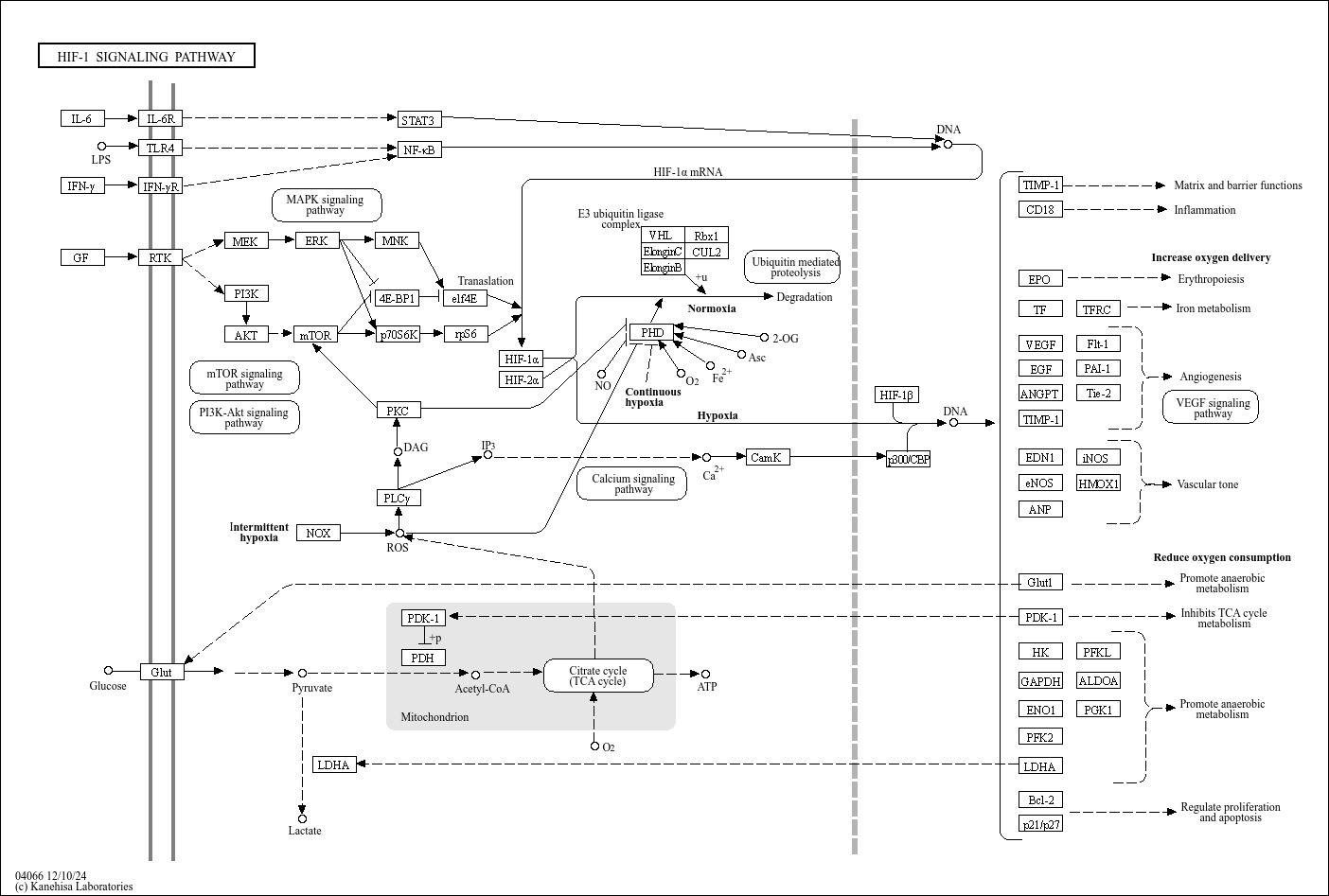


Figure 4: HIF-1 Signaling Pathway


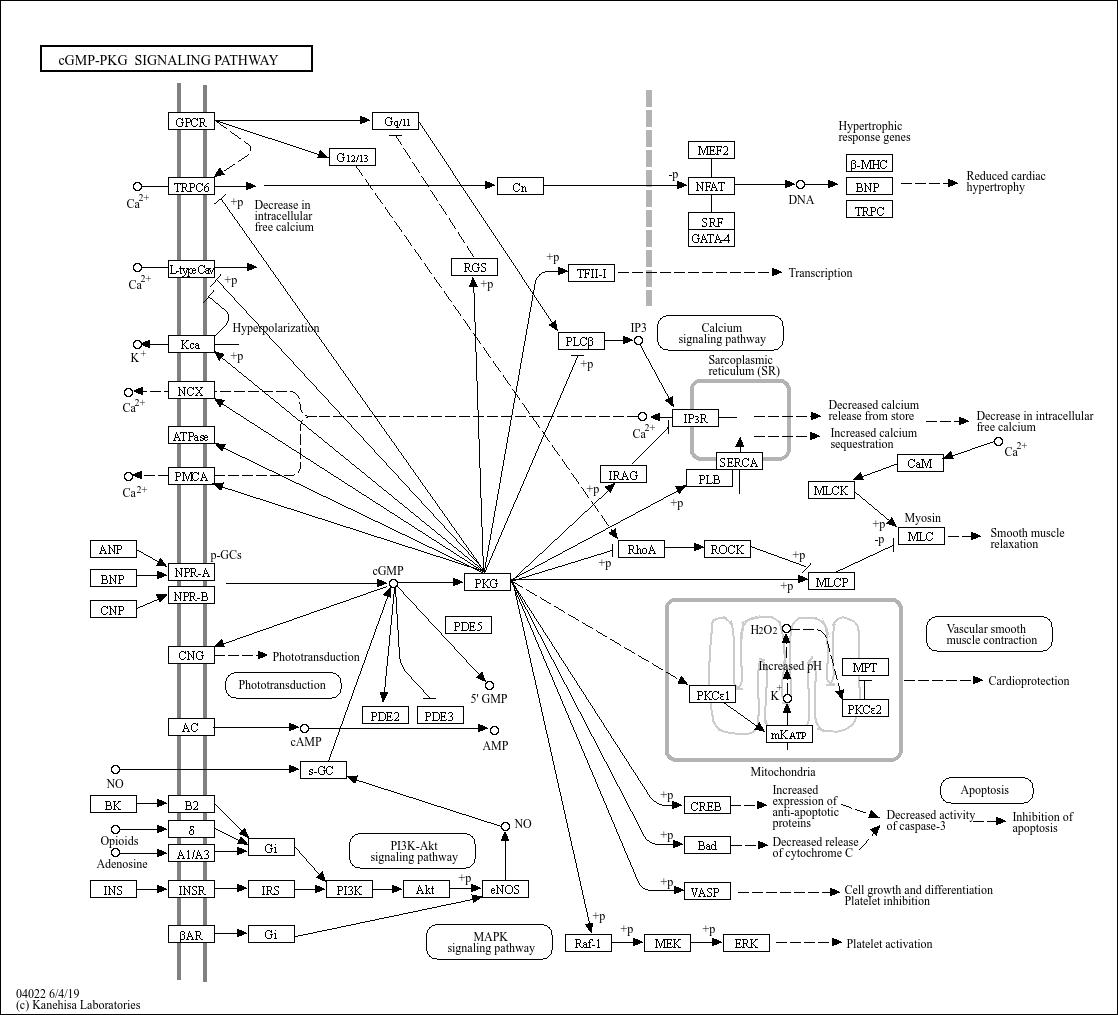


Figure 5: cGMP-PKG Signaling Pathway


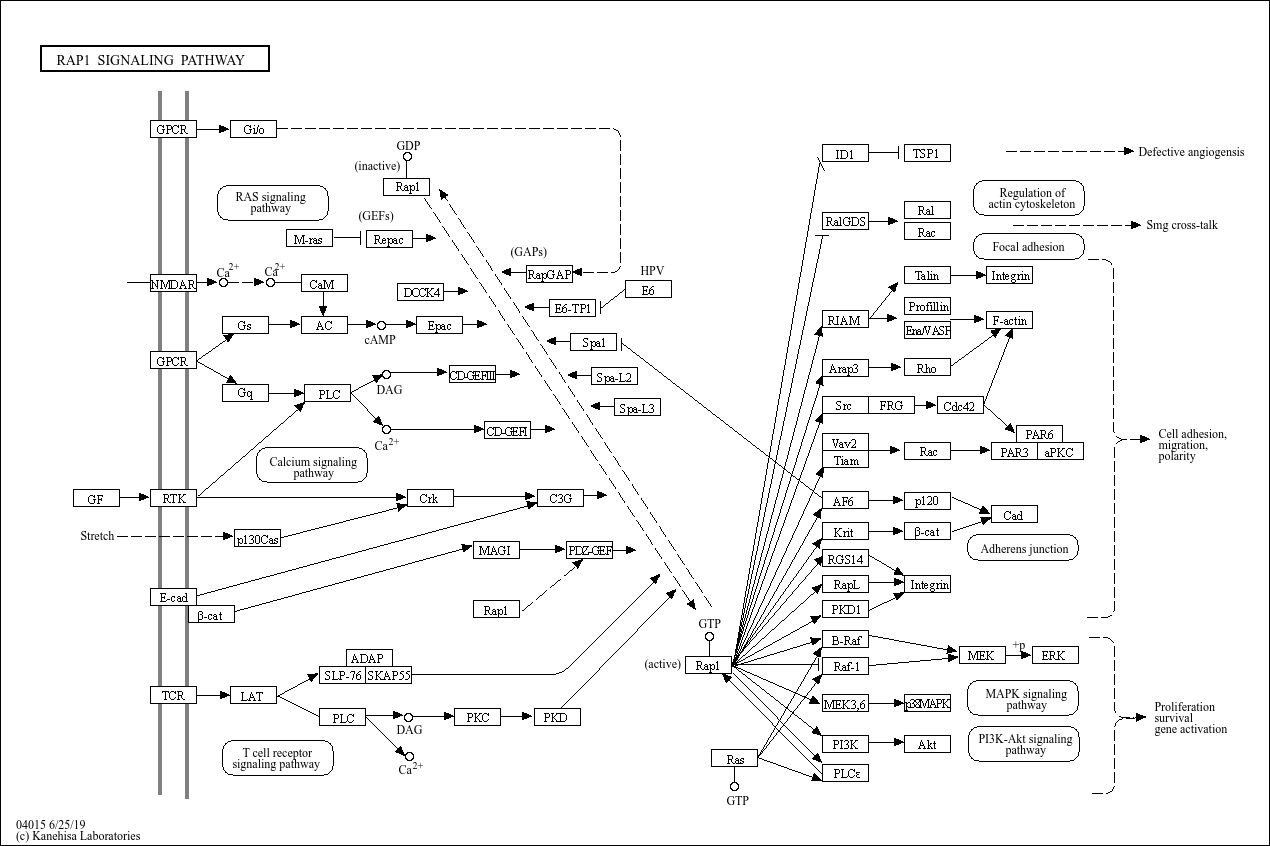


Figure 6: Rap1 Signaling Pathway


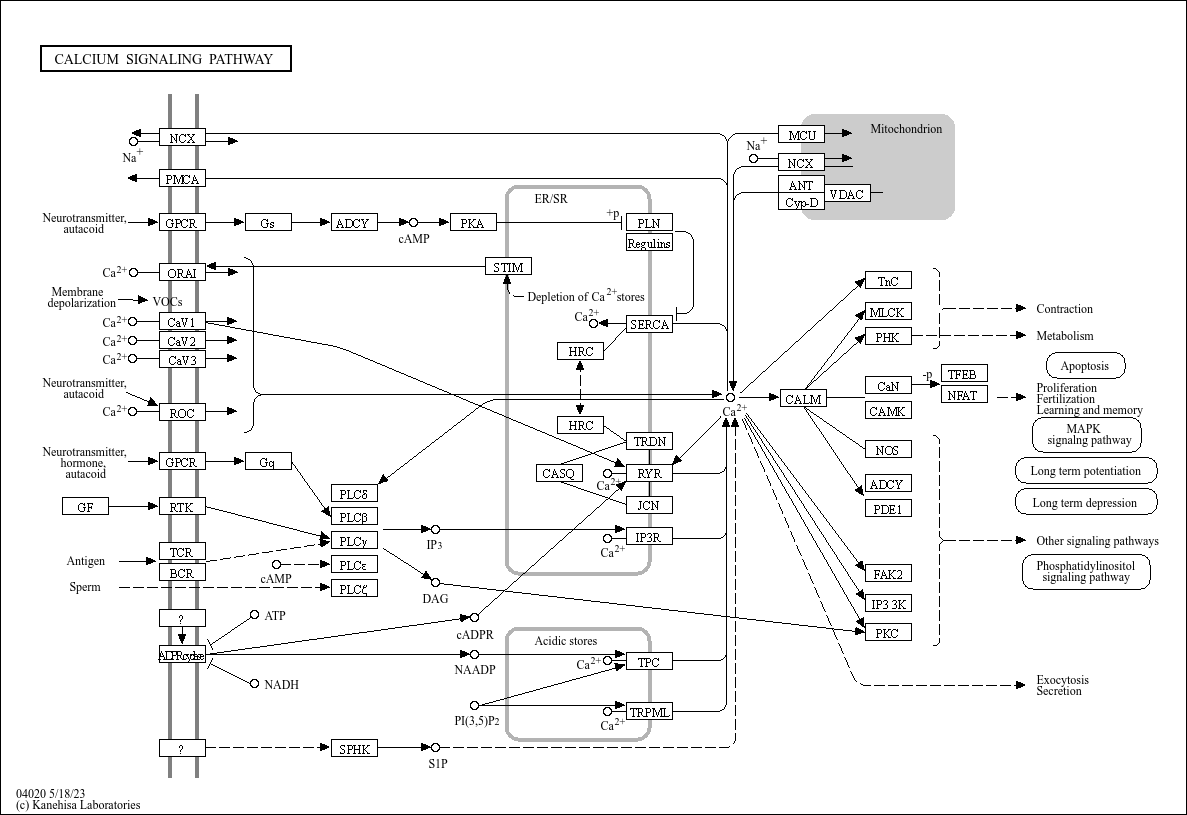


Figure 7: Calcium Signaling Pathway


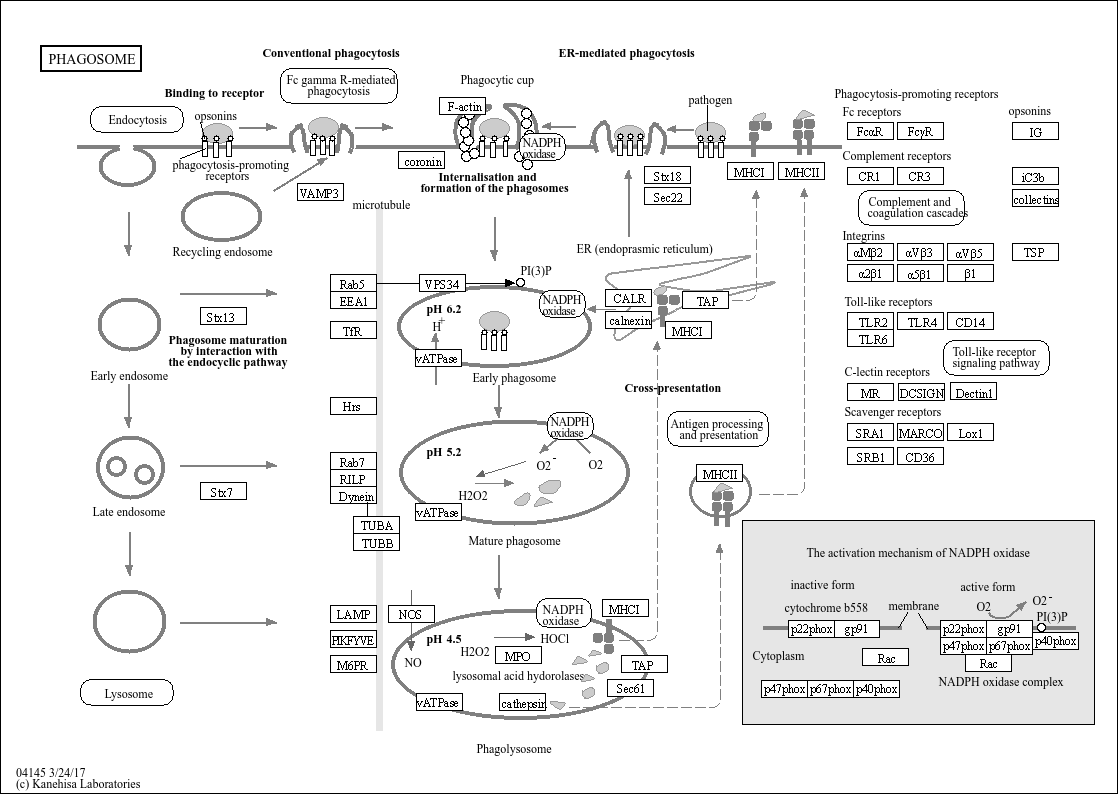


Figure 8: Phagosome Pathway


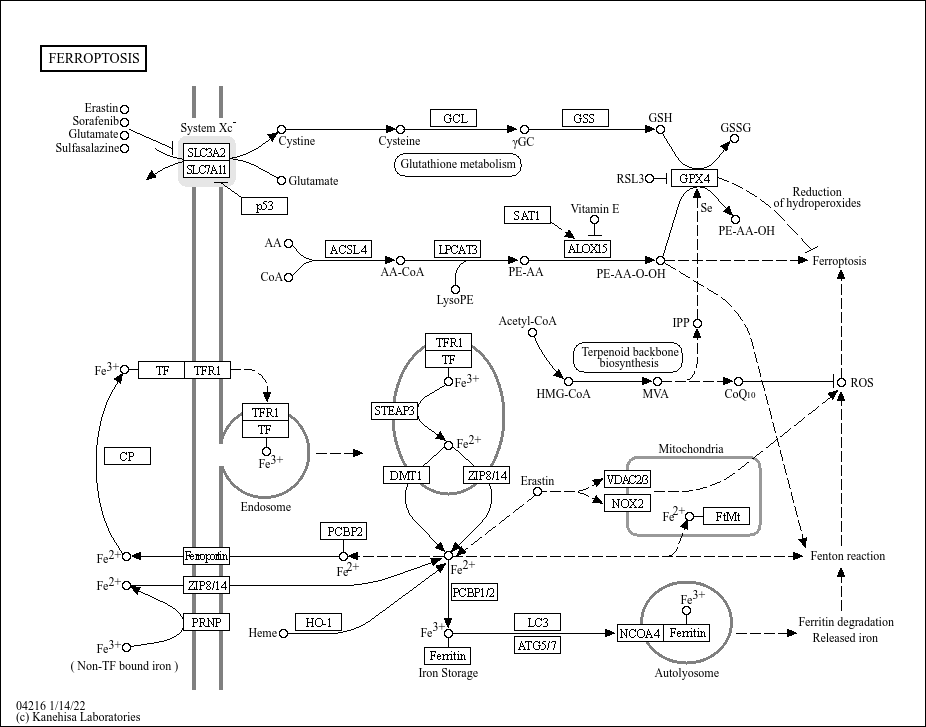


Figure 9: Ferroptosis Pathway


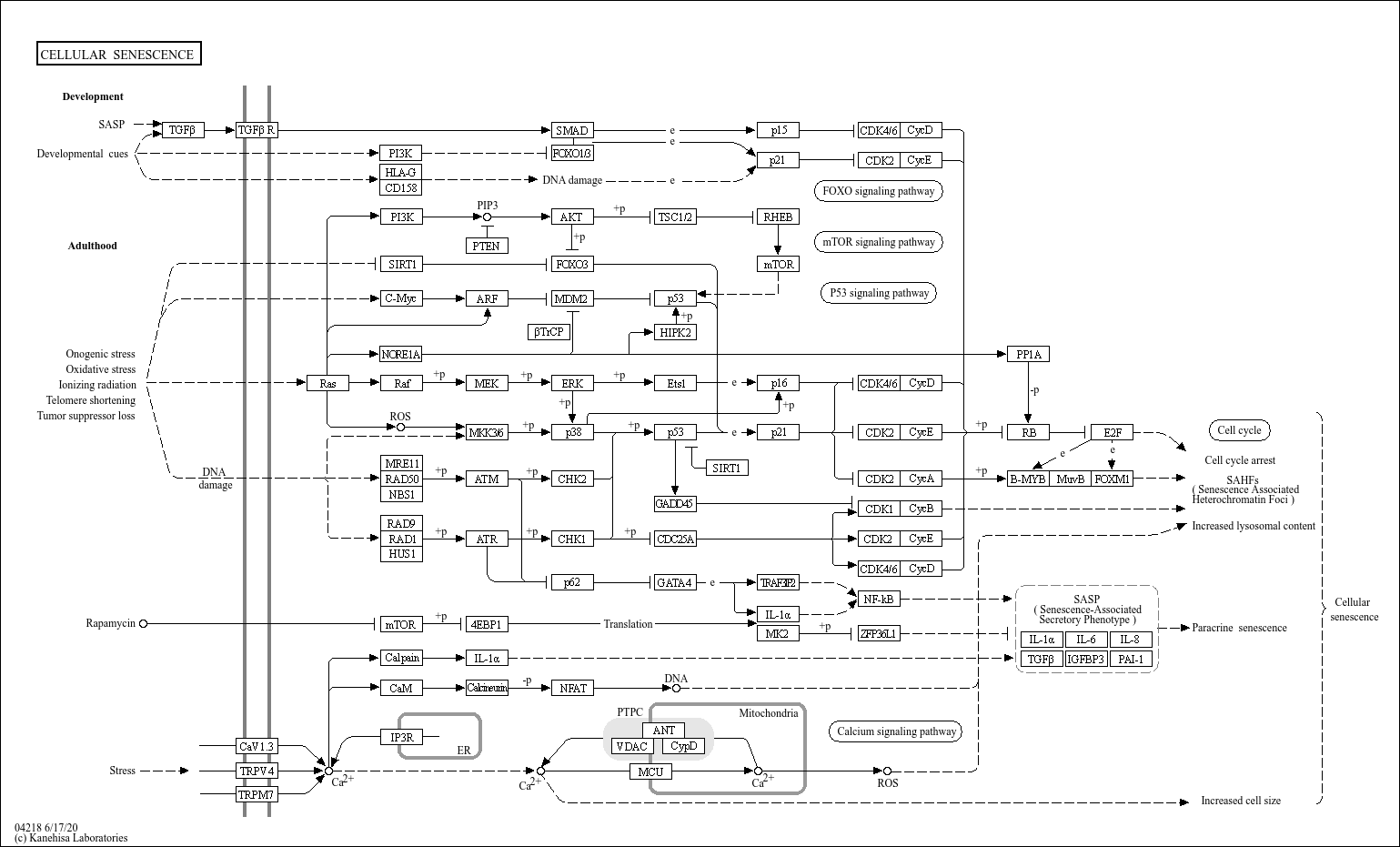


Figure 10: Cellular Senescence Pathway


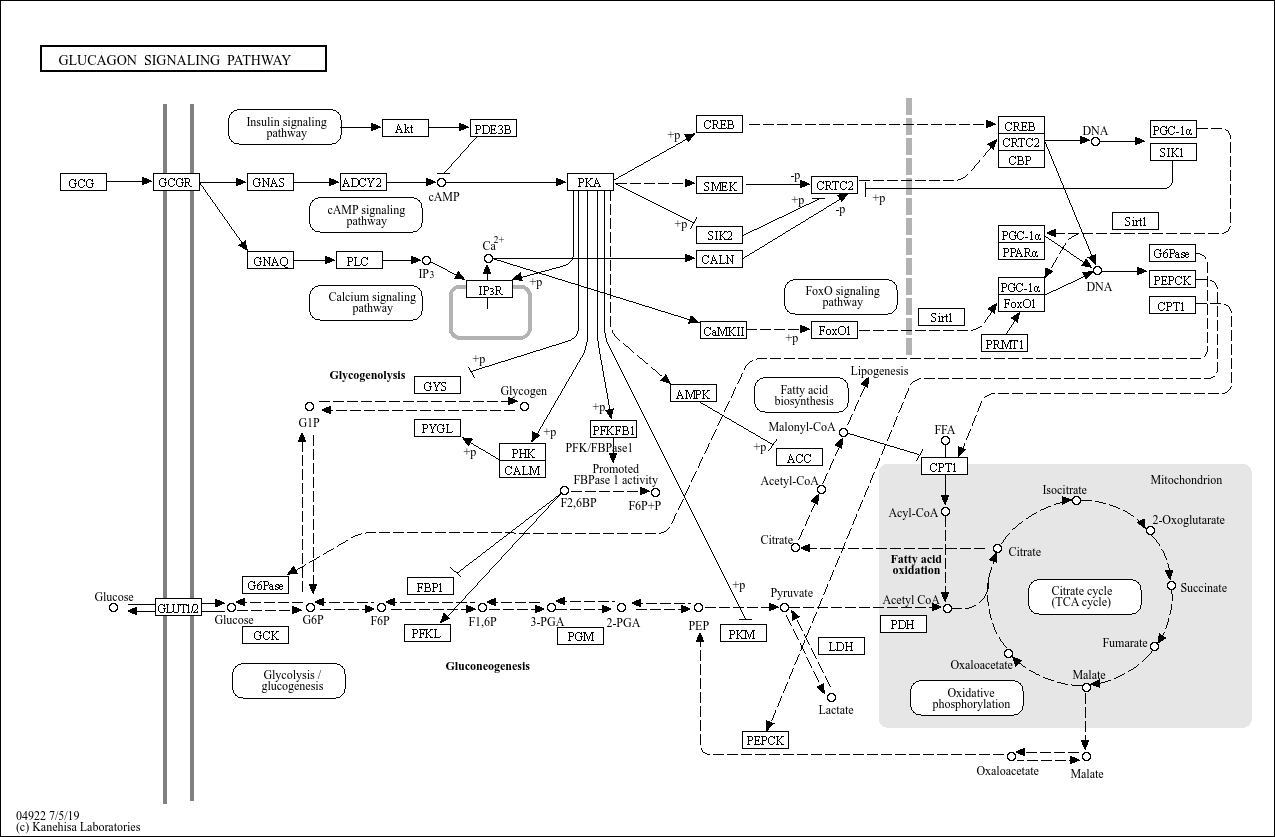


Figure 11: Glucagon Signaling Pathway


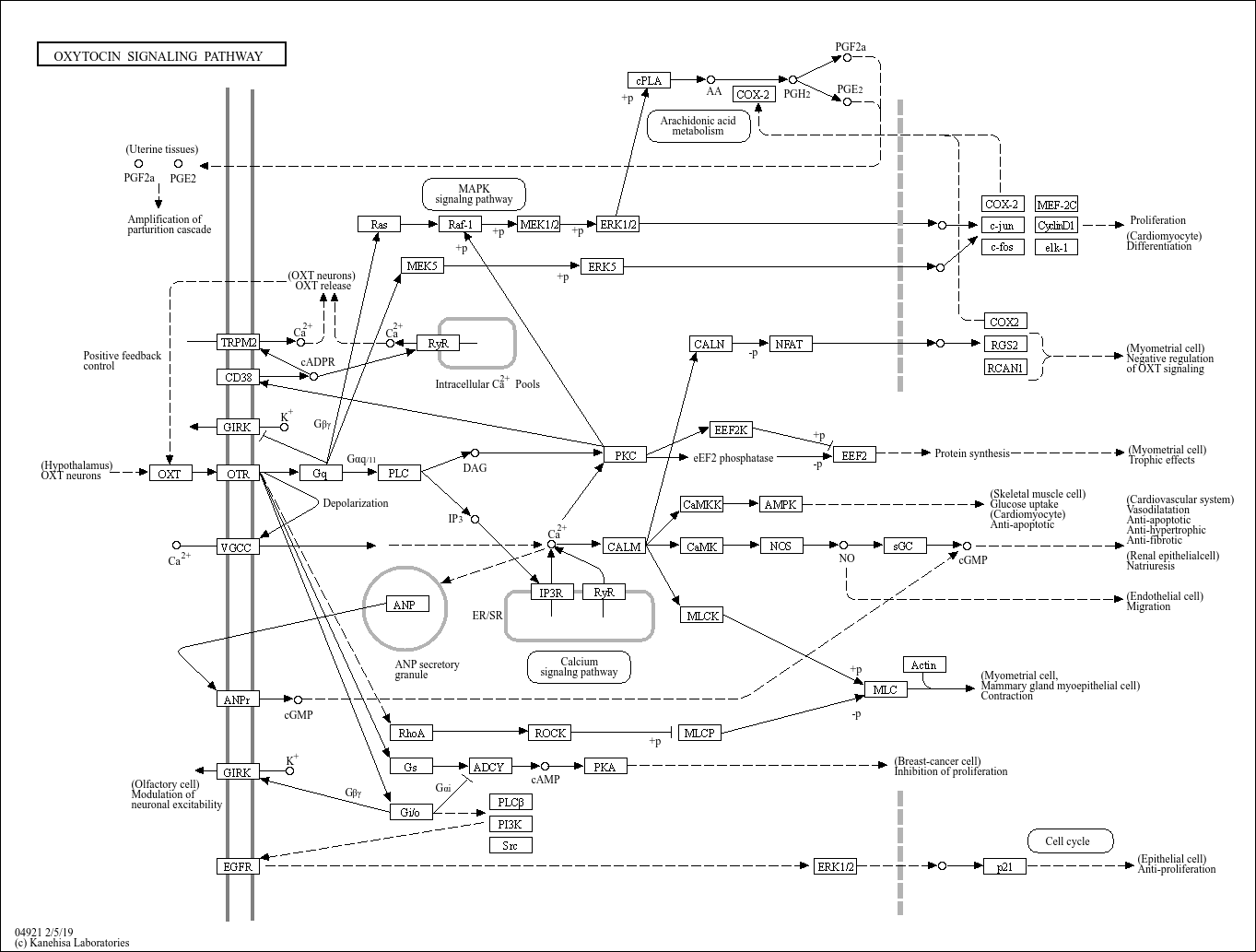


Figure 12: Oxytocin Signaling Pathway


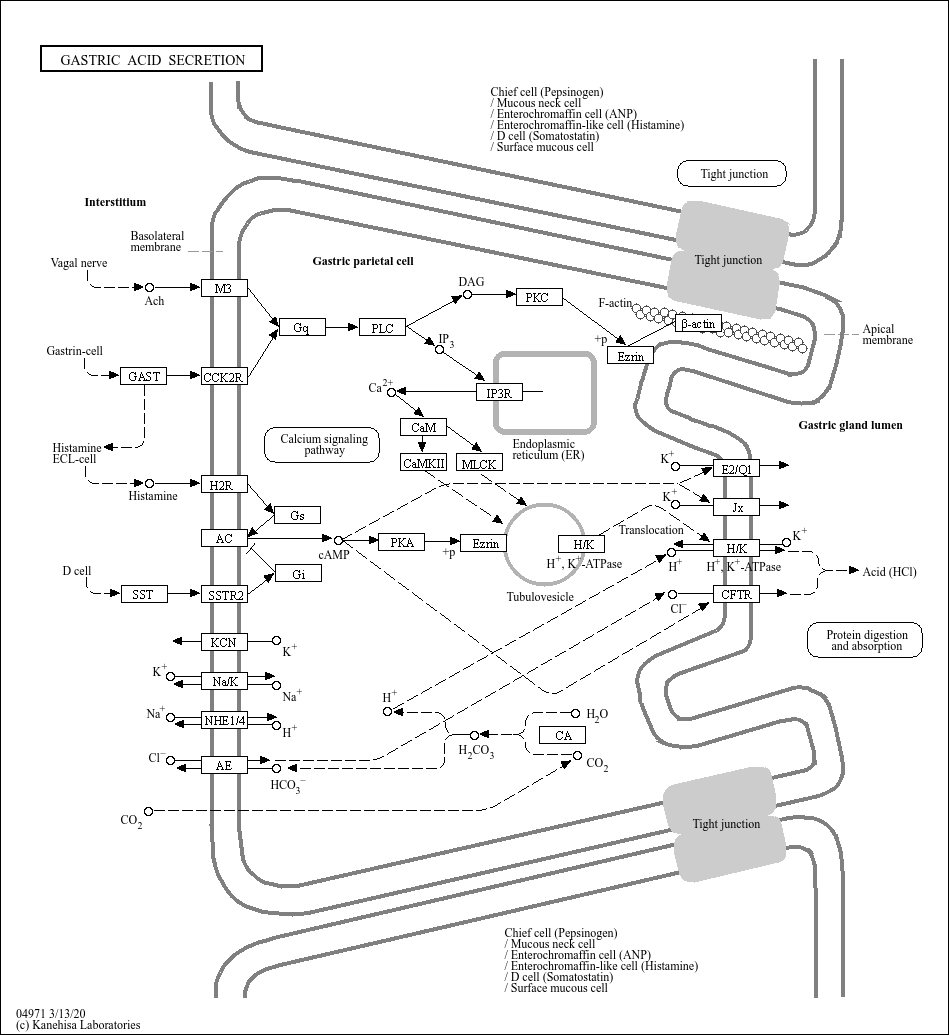


Figure 13: Gastric Acid Secretion Pathway
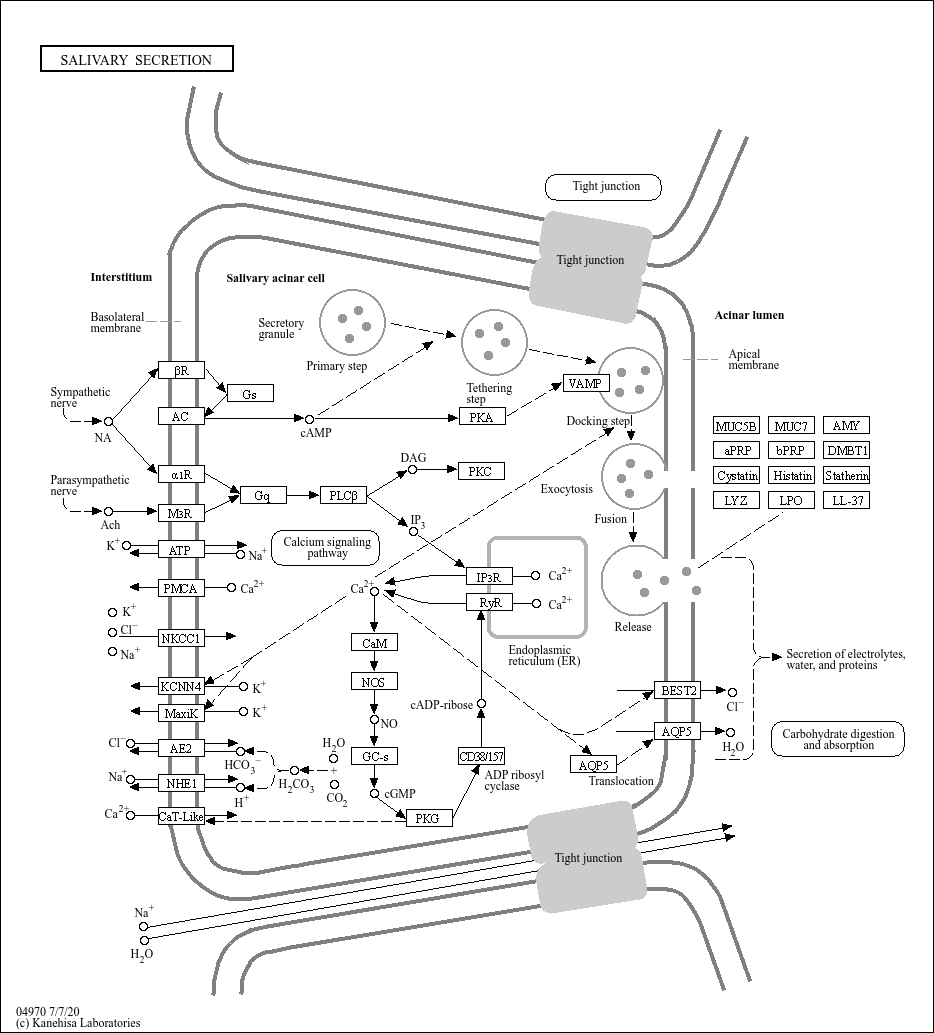


Figure 14: Saliva Secretion Pathway


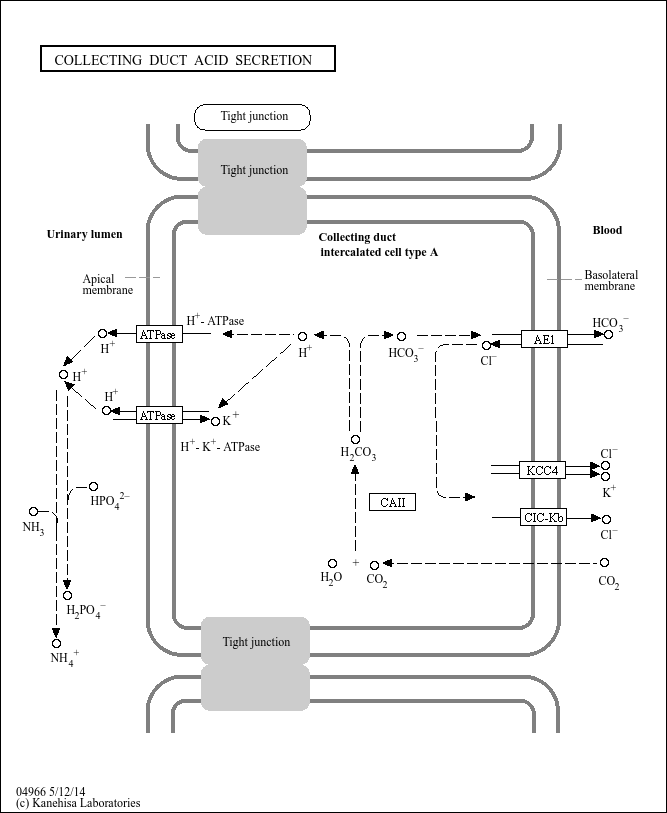


Figure 15: Collecting Duct Acid Secretion Pathway


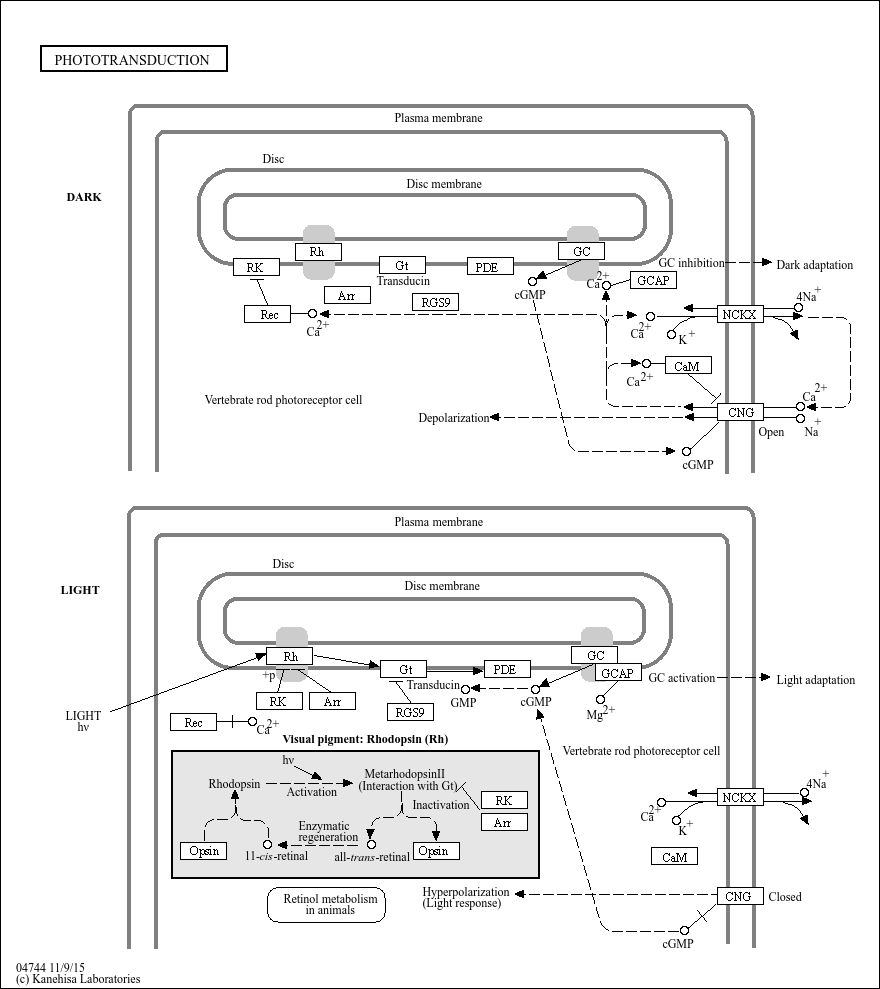


Figure 16: Phototransduction Pathway


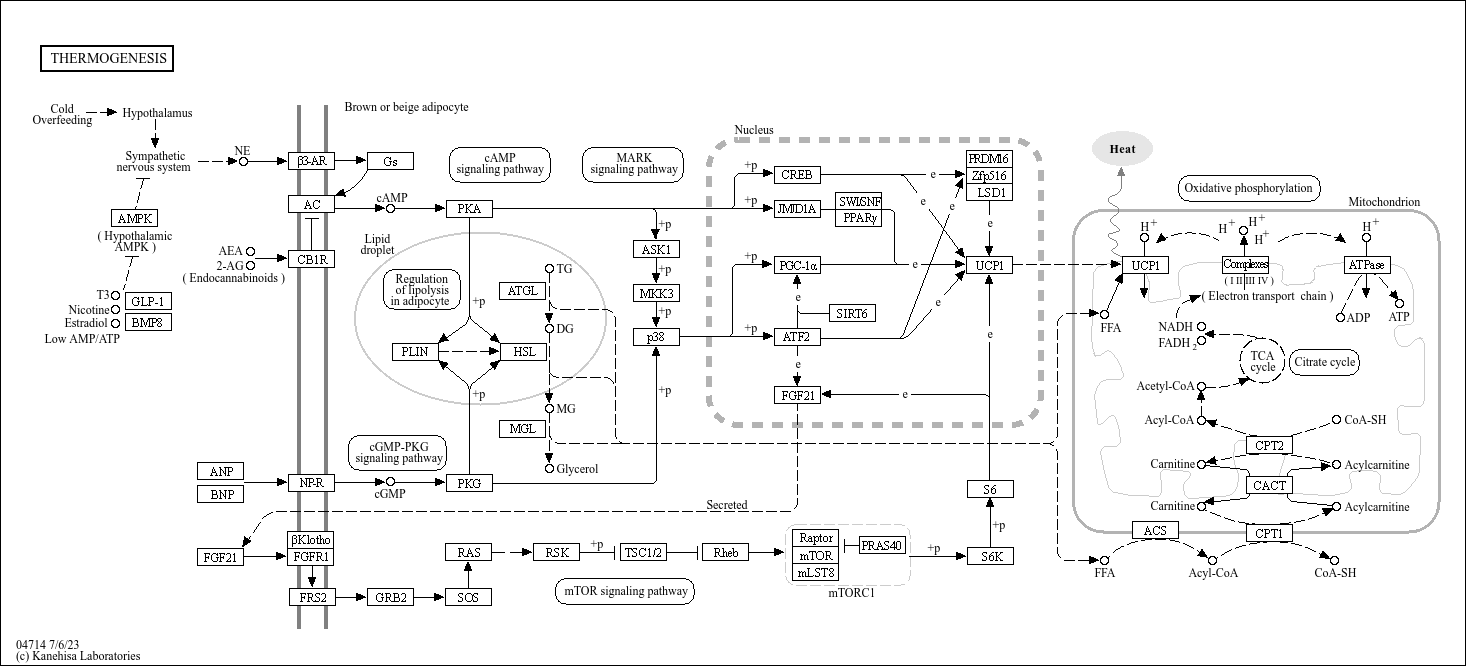


Figure 17: Thermogenesis Pathway
